# The medicinal plant used in the Guangxi Fangcheng Golden Camellias national nature reserve, a coastal region in southern China

**DOI:** 10.1186/s13002-023-00605-4

**Published:** 2023-07-27

**Authors:** Renchuan Hu, Kedao Lai, Binsheng Luo, Renjie Tang, Ruibin Huang, Xiaoxia Ye

**Affiliations:** 1 Guangxi Key Laboratory of Traditional Chinese Medicine Quality Standards, Guangxi Institute of Traditional Medical and Pharmaceutical Sciences, Nanning, 530022 China; 2 Lushan Botanical Garden, Jiangxi Province and Chinese Academy of Sciences, Lushan, 332900 China; 3 Guangxi Fangcheng Golden Camellias National Nature Reserve Management Center, Fangchenggang, 538021 China; 4 Bioengineering and Technology Center for Native Medicinal Resources Development, Yulin Normal University, Yulin, 537000 China

**Keywords:** Medicinal plant, Medicinal market, Ethnobotany, Traditional knowledge, Sustainability

## Abstract

**Backgrounds:**

Guangxi Fangcheng Golden Camellias national nature reserve, situated in Fangcheng City, Guangxi Province, China, is a coastal region renowned for its exceptional natural environment. Over time, the residents of this area have acquired extensive knowledge regarding medicinal plants, owing to their close association with the abundant flora. Our study aims to document the medicinal plants used by the local community near the Guangxi Fangcheng Golden Camellias national nature reserve. We seek to investigate the unique regional properties, cultural significance, and potential connections between medicinal plants used in surrounding villages and those sold in markets.

**Methods:**

During 2019–2021, 96 informants, including 36 key informants, were interviewed in the study area. The snowball sampling method was used to select respondents from medicinal markets and villages. Local therapists were defaulted as key informants. A panel discussion was held on the protection and threat of medicinal plants and traditional knowledge. In this study, two quantitative indicators, relative frequency citation (RFC) and informant consensus factor (ICF), were used to analyze the traditional medicinal plants in the study area.

**Results:**

According to the investigation, a total of 396 species of medicinal plants belonging to 295 genera and 116 families were recorded. From the perspective of Lifeform, herbs accounted for 38.9%, followed by shrubs. Most of the medicinal parts are whole plant (120 species, 25.59%), branches and leaves (116 species, 24.73%), and roots (101 species, 21.54%). Medicinal bath is the most commonly used therapeutic method. Among the 13 therapeutic targets recorded, rheumatic drugs accounted for the highest proportion, followed by muscular system diseases and skin-related diseases, which are closely related to local climate and livelihood. ICF shows that the use of local medicinal plants and related knowledge is very diverse, so local people have more options for treating diseases. *Melicope pteleifolia*, *Clerodendrum cyrtophyllum*, *Lygodium flexuosum, Elephantopus scaber, Artemisia argyi, Plantago asiatica, Centella asiatica, Grangea maderaspatana, and Liquidambar formosana* have high RFC, which are closely connected to local people's daily lives and are potentially vital to them. The wild vegetation, mostly around the nature reserve, is the primary source of medicinal materials sold in the urban medicinal market. Urban areas have fewer varieties of medicinal plants compared to villages near protected areas. However, there is consistency in their usage and application.

**Conclusion:**

The medicinal plants used in the villages near the Golden Camellia Nature Reserve are diverse, and the relevant traditional knowledge is relatively well preserved. The collection of medicinal materials by local people is sustainable. This study suggests that the local government should also protect relevant traditional knowledge in the decision-making process.

**Supplementary Information:**

The online version contains supplementary material available at 10.1186/s13002-023-00605-4.

## Introduction

The use of wild medicinal plants to treat diseases has always been a popular way of folk health care in many countries, especially in underdeveloped countries and remote areas [1, 2]. Herbal therapies differ from mainstream western medicine in that they often adhere to local theories and characteristics. While they may have scientific properties, many of these have not been verified by modern western science [3]. With the development of science and technology, the practice of traditional medical knowledge is facing the dilemma of being gradually replaced by western medicine because of its own limitations [2, 3]. Many cases have shown that the recording, mining, and scientific verification of traditional medical knowledge can better protect them and provide new ideas and clues for the research of modern therapy and new drugs [4].

As one of the four ancient civilizations, China also has a long history in traditional medicine. Up to now, many researchers have conducted rescue research on traditional Chinese and ethnic medicine, among which Guangxi Province is one of the hotspots [5]. For example, in recent years, some scholars have carried out research in the Dragon Boat Festival Medicine Market of Jingxi and Gongcheng in Guangxi, and more than 100 medicinal species have been recorded and analyzed [6, 7]. In the Luocheng area, Hu et al. also conducted a systematic investigation on the local medicinal plants of Mulam people and documented more than 400 species of medicinal plants [8]. These studies reveal the history and culture of medicinal herbs in Guangxi Province and the wealthy traditional knowledge of medicinal plants [6–8]. However, the investigation of medicinal plants and related pharmaceutical markets in the coastal areas of southern Guangxi is still blank.

**Fig. 1:**
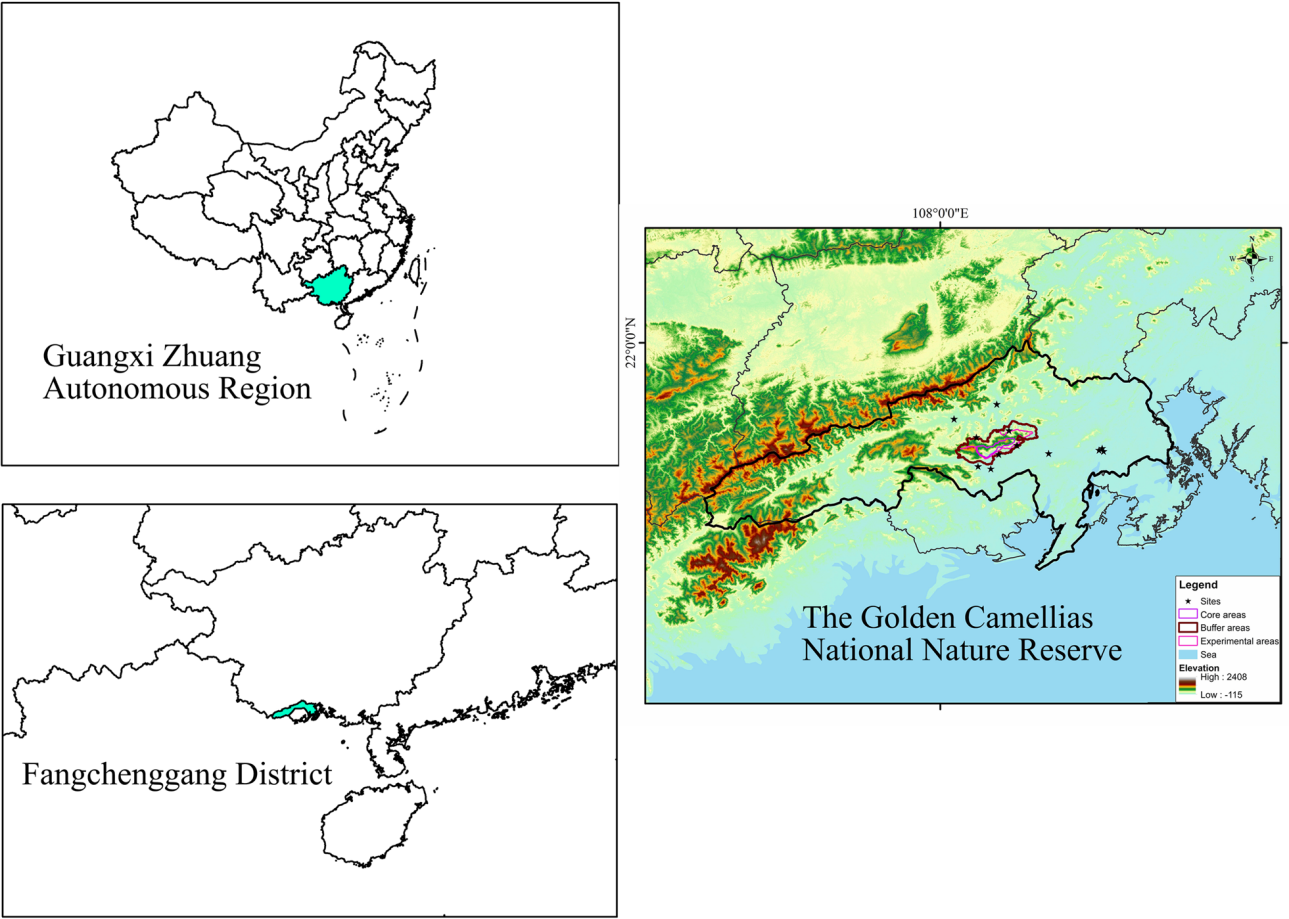
The location and topography of Fangcheng District and study sites

Fangcheng City is located at the confluence of the southern end of China's eastern coast and the southwest border, which is also on the coast of Beibu Gulf of Guangxi Province; it is one of the only two cities in China where the border and coast meet, with a total area of 6238 km^2^ [9]. It has jurisdiction over the Gangkou District, Fangcheng District, Shangsi County, and Dongxing City, with a population of about 1 million [9]. There are 21 ethnic groups in Fangchenggang, including Zhuang, Yao, and Kinh, the three main ethnic minorities [9]. The topography of Shiwan Mountain intercepts a large amount of water vapor blowing from the seaside, so Fangcheng city in the south has more rainfall, becoming one of the regions and rainstorm centers with the most rainfall in Guangxi, even the whole country [9] (Fig. 1). Relying on such an excellent natural environment and rich biodiversity, the central government established the Guangxi Fangcheng Golden Camellias national nature reserve in the Fangcheng area in 1994 [9]. The suitable eco-geographical environment has created a wealth of local plant species diversity. According to the latest census, about 1511 species of higher plants in Camellia National Reserve are belonging to 200 families and 773 genera (not yet published), and approximately 76.77% are medicinal species, which has exceptionally high protection value. Rich plant resources have laid a good material foundation for the inheritance and development of traditional medicine.

Over the national nature reserve, the local hot and humid climate has brought many complex and complicated diseases to the people, such as rheumatism, rheumatoid arthritis, skin diseases, etc. While fighting against the condition for a long time, the locals have explored and summed up a unique mode of disease prevention and treatment by using medicinal herbs, which are safe, effective, and low cost, like having baths and substitute tea. During our initial research on medicinal plants in the Fangcheng region, we discovered a thriving trade in these herbs. Based on our preliminary visiting, we found that a significant proportion of these plants were procured from the natural surroundings of the national nature reserve, rendering them immensely valuable for subsequent research. However, it has come to our attention that in the urban areas of Fangcheng District, conventional medicinal markets are also offering medicinal herbs that may potentially originate from the national nature reserve.

Our primary objective is to comprehensively record and document the utilization of medicinal plants by the indigenous community residing in proximity to the Guangxi Fangcheng Golden Camellias national nature reserve. Through our research, we aspire to investigate and analyze the distinctive regional characteristics and cultural importance associated with these plants. Furthermore, we endeavor to explore potential correlations between the medicinal plants employed by local villages surrounding the nature reserve and those available for sale in the markets in urban area in Fangcheng.

## Methods

### Study area

Fangcheng District is located on the bank of Beibu Gulf of China, located in the south of Shiwan Mountain, connected with the mountains and rivers of Vietnam. With a total area of 2427 km^2^, the region has jurisdiction over ten towns, three streets, 145 administrative villages, and 20 communities, with a total population of 452,000, mostly Han, Zhuang, and Yao ethnic groups. The region has a subtropical monsoon climate, abundant rainfall, a long frost-free period, and healthy and thick soil. The coastline is 141.7 km long, the shallow tidal flat area is 77.66 km^2^, there are many harbors, the water quality is excellent, and the marine resources are rich. The forest area of the region is 1 604.67 km^2^, and the forest coverage rate is as high as 65.99% according to a governmental report by Fangcheng District Government Office in 2021 (http://www.fcq.gov.cn/gk/fcqgk/202003/t20200319_97499.html).

According to the distribution characteristics of the local villages and the suggestions of the local government officials, we mainly selected two types of survey sites: one is the villages around the Nature Reserve, including Mizhong Village (108.1517° E, 21.8065° N, 68 m a.s.l.), Napai Village (108.0798° E, 21.7916° N, 138 m a.s.l.), Dalu Community (108.1246° E, 21.8644° N, 70 m a.s.l.), Huashi Community (108.2392° E, 21.7557° N, 44 m a.s.l.), Nawan Village (108.1694° E, 21.7738° N, 59 m a.s.l.), Naqin Community (108.0306° E, 21.8319° N, 127 m a.s.l.), Nasuo Community (108.1116° E, 21.7217° N, 20 m a.s.l.), Nafu Village (108.1292° E, 21.7539° N, 42 m a.s.l.), and Pingmu Village (108.0835° E, 21.7273° N, 36 m a.s.l.); the other is the herbal stalls in the densely populated area (urban area) of Fangcheng District, including Jiangbin market (108.3502° E, 21.7629° N, 25 m a.s.l.), Zhongxin market (108.3571° E, 21.7667° N, 26 m a.s.l.), and Erqiao market (108.3604° E, 21.7603° N, 24 m a.s.l.) in Fangcheng downtown area (Fig. 1).

### Data collection

From December 2019 to November 2021, a comprehensive study was conducted in the designated area, involving interviews with a total of 96 informants, of which 36 were identified as key informants. The snowball sampling method was employed to strategically select participants from diverse backgrounds, including local therapists, herbal shop owners, medicine collectors, village cadres, and other relevant individuals within the medicinal markets and villages (communities). Based on the snowball sampling selection, local therapists were specifically designated as key informants due to their prominent role in possessing and transmitting invaluable native medicinal plant knowledge [10]. Before each interview, informed consent was required, and international ethics were observed throughout the study. The information about plant medicine was collected utilizing semi-structured interviews, participatory observation, and field investigation [11, 12].

During the interview with informants, the local names of plants, the medicinal purposes, the medicinal parts, and the preparation methods were recorded in detail. Among them, for the classification of diseases treated by plants, we refer to the local folk classification of conditions and the International Classification of Diseases [13, 14]. In addition, a panel discussion was held on the protection and threat of medicinal plants and traditional knowledge.

We also collected herbarium voucher specimens and took photos during the survey as records [15], and all specimens were preserved in the Herbarium of the Guangxi Zhuang Autonomous Region, Institute of Traditional Chinese Medicine (GXMI). The *Flora of China*, Flora of Guangxi (Guangxi Institute of Botany, 2005), and botanical websites (e.g., www.worldfloraonline.org., http://www.cvh.ac.cn/search, http://ppbc.iplant.cn/) were used assisting the taxonomic identifications.

### Quantitative analysis

In this study, two quantitative indicators, relative frequency citation (RFC) and informant consensus factor (ICF), were used to analyze the traditional medicinal plants in the study area.

The RFC was calculated without considering the use categories by following the formula [12, 16]:

$${\text{RFC}} = \frac{{{\text{FC}}}}{N}\quad (0 < {\text{RFC}} < 1)$$

RFC shows the importance of each species in the study area given by the FC divided by *N*; FC is the number of local informants who reported the uses of a specific plant species or the number of species appearing on market stalls; The *N* means the total number of informants or market stalls. The higher the RFC, the more frequently it is used in the local area, which may be more closely related to the daily life of the local people [12, 16].

Twelve ailment categories were identified based on the eight systems of the human body and drug characteristics of local people, and the informant consensus factor (ICF) was calculated to determine the effectiveness of medicinal plants in each ailment category [17, 18]. The formula is listed below:

$${\text{ICF}} = \frac{{{\text{nur}} - {\text{nt}}}}{{{\text{nur}} - 1}}$$

Nur means the number of individual plant use reports for a particular illness category, while nt means the total number of species used by all informants for this illness category [17, 18].

## Results

### Basic characteristics of local traditional medicinal plants

According to the investigation, we recorded a total of 396 species of medicinal plants belonging to 116 families and 295 genera (see Additional file 1: Table S1). In the family distribution, Fabaceae has the most significant number of species, including 29 species, followed by Asteraceae and Rubiaceae, both with 21 species; In terms of genera, *Ardisia* contains the most considerable number of species, including eight species, followed by *Bauhinia* and *Ficus*, containing with six species and five species respectively. Among the recorded herbs, 22 families include more than five species, with 235 species, accounting for 59.34% of the total species; 44 families contain 2–4 species, with 111 species, accounting for 28.03%, while 50 single-species families account for 12.63%. At the genus level, there are three genera with more than five species, with 19 species, accounting for 4.80% of the total species; 44 genera with 2–4 species, with 150 species, accounting for 37.88%, and 227 single-species genera, accounting for more than half of the whole species (57.32%).

Generally speaking, the medicinal plants used around the Guangxi Fangcheng Golden Camellias national nature reserve are mainly concentrated in multi-species families, which is the cluster distribution at the family level. In contrast, these plants are primarily distributed in single-species-genera and scattered at the genus level. It reflects that the residents in this area have diversity in the use of medicinal plants, and the relevant traditional knowledge is prosperous.

As for the life forms, among these traditional medicinal plants, herbs account for 38.9%, shrubs take second place, accounting for 26%, and trees and vines account for 17.7% and 17.4%, respectively. More than 93% of the medicinal plants used in this area come from the wild environment around the Guangxi Fangcheng Golden Camellias national nature reserve (369 species). There are only 27 cultivated species, most of which have local uses other than medicine. For example, some cultivated plants are mainly used for food, including *Citrus maxima*, *Clausena lansium*, *Dimocarpus longan*, etc., and some plants are used mainly for ornamental purposes, such as *Agave sisalana* and *Alstonia scholaris*.

### Medicinal part

**Fig. 2:**
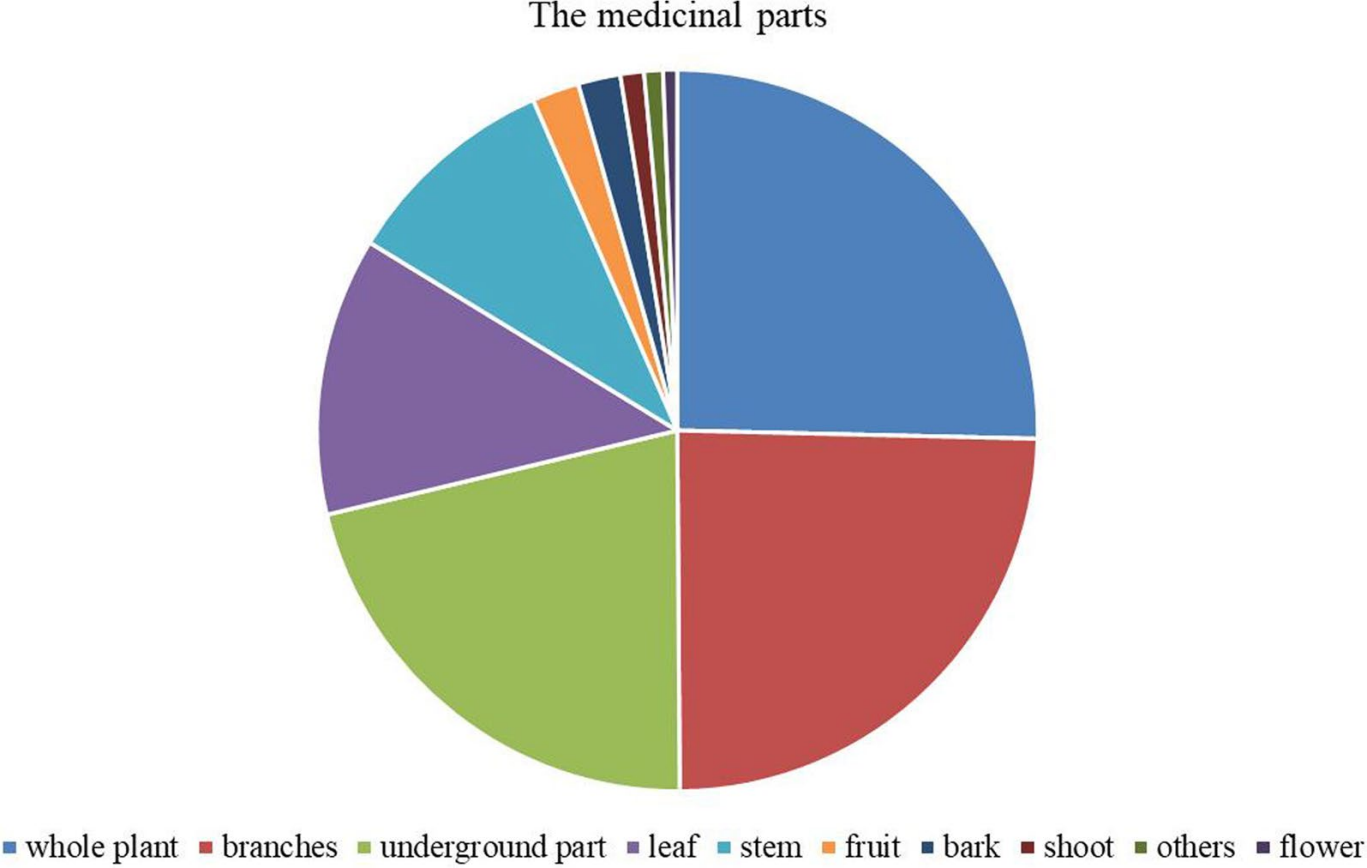
The medicinal parts of recorded plants

The medicinal parts of the recorded plants were divided into ten categories: whole plant, branches, underground part (root, tuberous root, rhizome, tuber), leaf (old leaf, tender leaf), stem (vine, transverse stem, stem pulp, old stem, bulb), fruit (seed, fruit, pericarp, seed, melon pulp), bark (root bark), shoot, flower (flower bud, inflorescence), other (camphor, sap, gum, nutrition bulb) and so on. As shown in Fig. 2, the use of whole plant (120 species, 25.59%), branches and leaves (116 species, 24.73%), and roots (101 species, 21.54%) as medicinal parts accounted for the vast majority, significantly higher than other types. As the whole plant is used as the largest proportion of medicinal plants, which contains a large number of herbaceous plants, the general herbaceous plants are small, and their use is often in the form of whole plants. In addition, the higher frequency of utilization of the entire plant, branches, and leaves is also related to the fact that local people like to use plants for external medicinal bathing. The underground part also accounts for more because, according to the local tradition, the root of the plant, especially the root tuber, is considered to be the most effective part, and the local people like to give priority to the use of the root, and its primary use is often decoction or making medicinal wine. Among the recorded plant species, there are also many uses and multiple utilization parts of one plant, reflecting the rich knowledge of local medicinal plants.

### The diseases targeted by medicinal plants

**Fig. 3:**
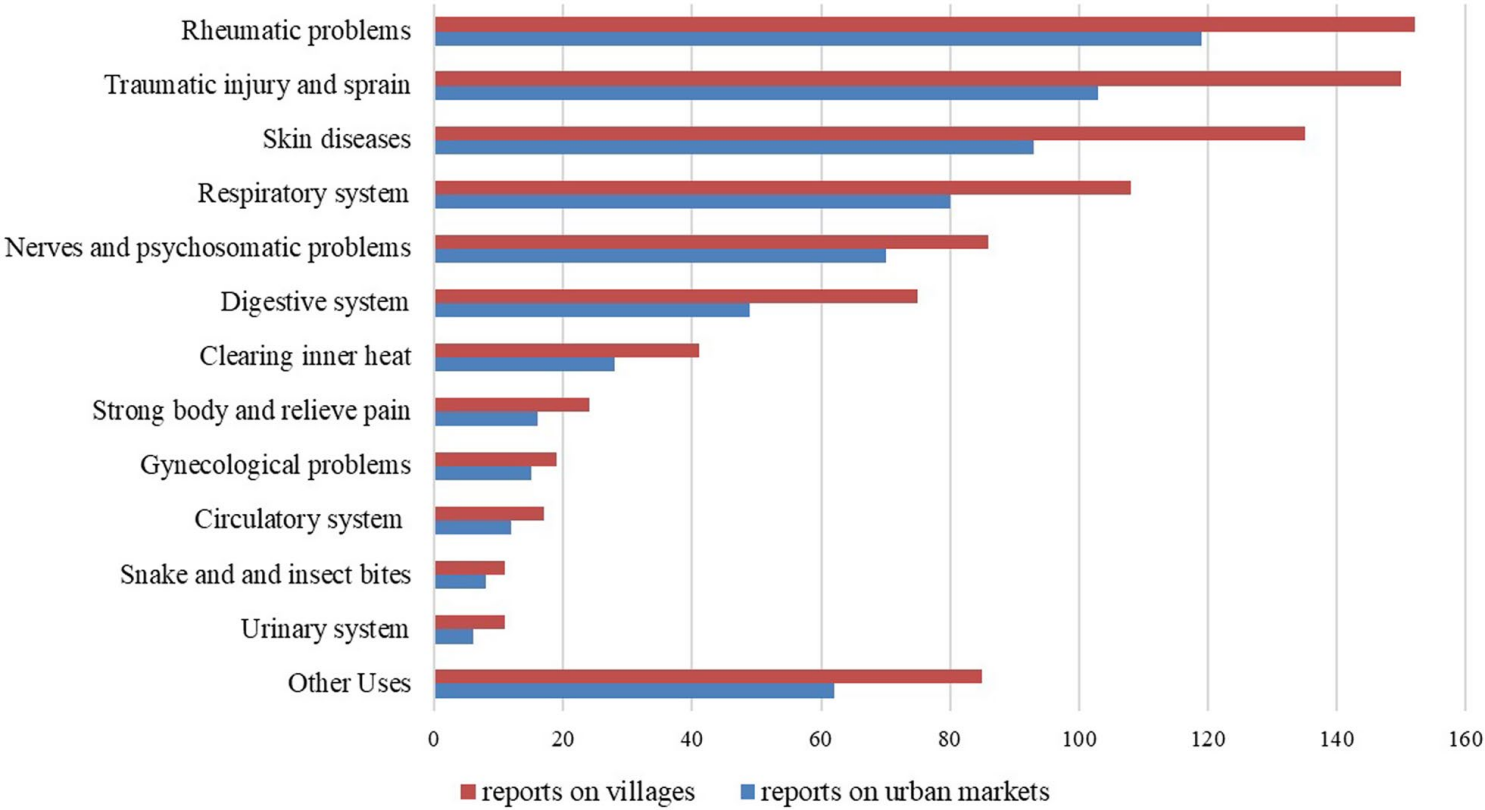
The diseases targeted by medicinal plants

**Fig. 4:**
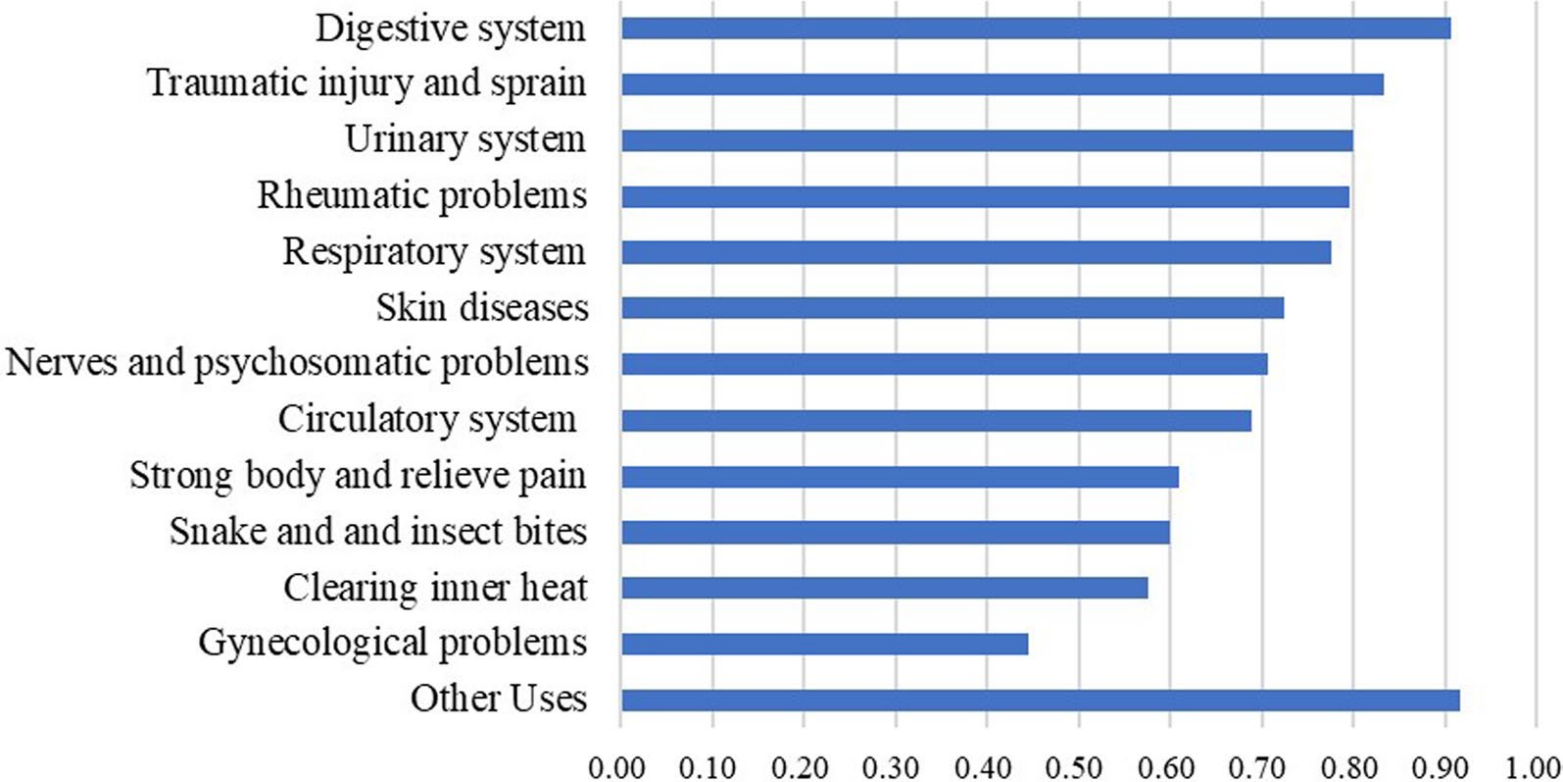
The ICF value of diseases targeted by medicinal plants

The diseases treated by medicinal herbs locally are divided into 13 categories, as shown in Figs. 3 and 4 [19]. After analysis, we found that in terms of the types of diseases targeted by medicinal plants, the trend of medicinal plants sold in urban areas was consistent with that of plants investigated in villages around the reserve. Combined with the interview's contents, we found that the herbs sold by urban medicine shops were mainly purchased from local medicine collectors, and these wild medicinal plants were collected around the nature reserves. This is probably one of the main reasons why medicinal plants in both urban areas and villages target similar diseases.

In addition, according to Fig. 3, herbs for rheumatism account for the highest proportion, followed by muscle system diseases and skin-related diseases, which are closely related to the local climate and livelihoods. As the local people are located in the mountain areas, the daily livelihood is primarily manual labor, which is very heavy; coupled with the long-term hot and humid local climate, rheumatism, muscle system diseases, and skin diseases are prevalent. In addition, herbs for treating respiratory diseases also account for a high proportion (50 species), especially those for cough. In the coastal areas of Fangcheng, the rainy season is very long, during which the weather in the rainy season changes significantly; the alternation of hot and cold temperatures particularly easy to causes colds, a major cause of cough. The rainy season is a period of frequent coughing, which could further cause many bronchial diseases.

The value of ICF is in the range of 0–1. The greater the ICF value, the more significant difference in the use of plant species when treating a specific disease; otherwise, it is more concentrated. According to our analysis (Fig. 4), the ICF values of all the medicinal plants we recorded are higher than 0.6. The ICF shows that the use of local medicinal plants and related knowledge is very diverse, so the local people have more choices in treating diseases. Among them, the species for treating gynecological diseases are relatively concentrated and consistent. However, in this study, we have recorded fewer amounts of plant species to treat gynecological ailments, which may also be related to the lower ICF.

### Usages

The methods of use are divided into four categories: therapeutic diet, internal administration, medicinal bath, and external use. Among the methods of use, the proportion of boiled water washing (medicinal bath) is the highest, the local climate is hot all year round, and the rainfall is abundant, which provides ideal conditions for the growth of local plants but also brings dampness and poison to the residents. The medicinal bath for disease prevention and treatment formed by the villagers around the natural reserve is a relatively safe and convenient form that belongs to a prominent local feature and has significant research value. Moreover, the medicinal bath has a good effect on muscle relaxation and treating rheumatism and skin diseases. Therefore, in the analysis of the usage of medicinal plants, medicinal bath accounts for the highest proportion, which also has a lot to do with the local natural environment and the physical livelihood of the local people.

In addition, there is a high proportion of decoction, which tends to deal with more diverse diseases, such as digestive diseases, metabolic diseases, etc. Similarly, the ratio of external use is also very high, and plants are usually mashed and applied to the affected area, which is generally used to treat skin diseases. The frequent occurrence of skin diseases also has a lot to do with the hot and humid climate of the area. On the other hand, diet therapy is the least, generally used for brewing wine or cooking directly (Table 1).

**Table 1:** The species number of usage in villages and urban markets

Usages	Species no. on villages	Species no. on urban markets
Therapeutic diet	20	9
Oral administration	182	112
Medicinal bath	218	162
External application	113	63

### Relative frequency citation (RFC)

**Fig. 5:**
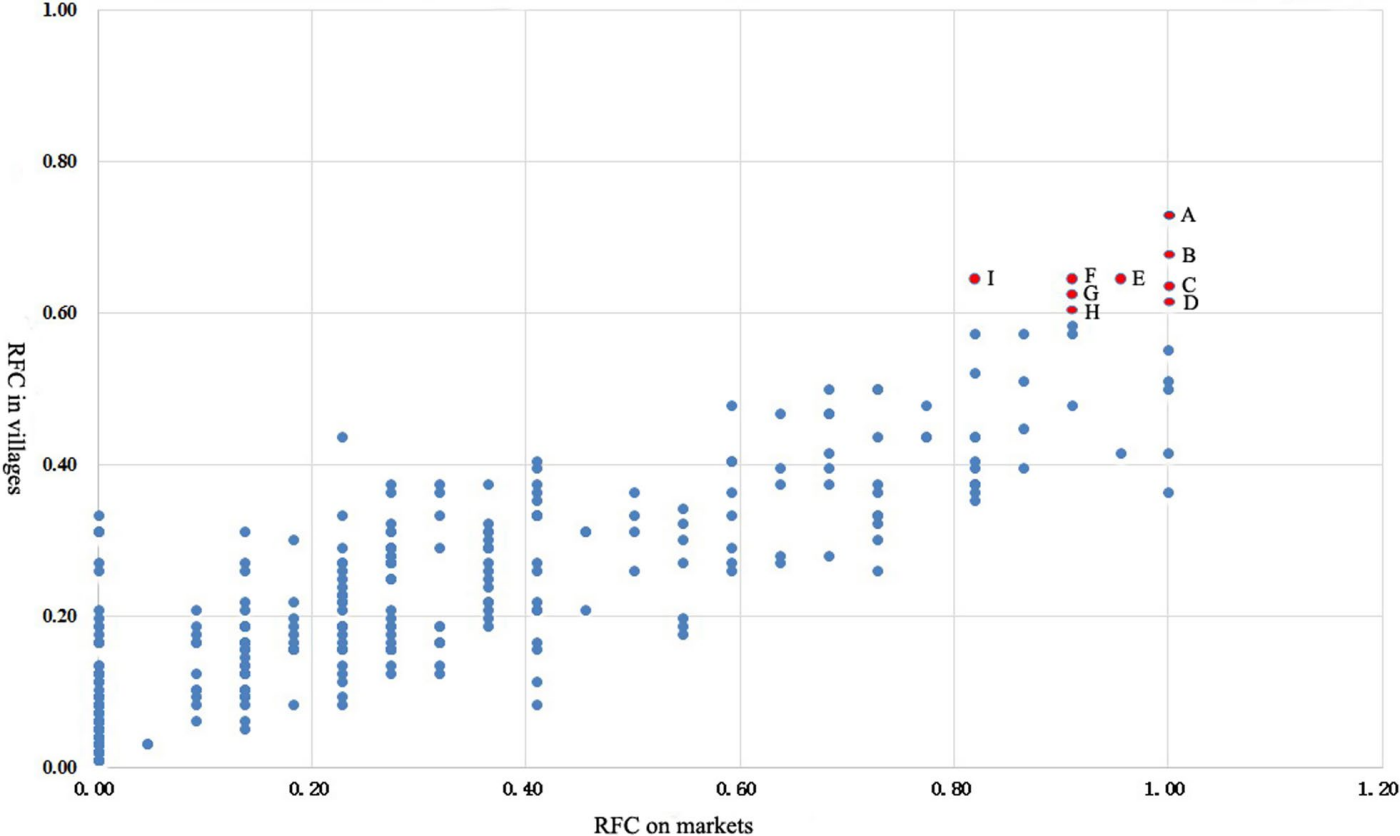
The RFC values of recorded medicinal plants (A. *Melicope pteleifolia*, B. *Clerodendrum cyrtophyllum*, C. *Lygodium flexuosum*, D. *Elephantopus scaber*, E. *Artemisia argyi*, F. *Plantago asiatica*, G. *Centella asiatica*, H. *Grangea maderaspatana*, I. *Liquidambar formosana*)

The RFC indicator is used to see how potentially important the plant species is for local people. Some plants have high RFCs in both urban areas and villages, including *Melicope pteleifolia, Clerodendrum cyrtophyllum, Lygodium flexuosum, Elephantopus scaber, Artemisia argyi, Plantago asiatica, Centella asiatica, Grangea maderaspatana, Liquidambar formosana* (Fig. 5). The plant species we listed above are closely connected to local people's daily lives and are potentially vital to them. In addition, those high-RFC plants grow widely in local wild environments and with large biomass. The local people's choice of herbal medicine is also scientific. For example, the plants with high RFC in this study are typical and famous ethnic medicine and also appear in some other ethnobotanical cases, such as the most common medicinal herb, *A. argyi* [20]. There are also many phytochemical and pharmacological studies to verify the efficacy of these plants, such as *M. pteleifolia*, which has the highest RFC [21]. Pharmacological studies have shown that crude extracts from *M. pteleifolia* exhibit anti-inflammatory, antibacterial, anti-tumor, liver protective, antihyperlipidemic, and hypoglycemic effects [21].

## Discussion

### The difference and connection between urban areas and villages

According to the survey, the plants found in the herbal stalls in urban markets were all included in the village survey, accounting for about 60% of the total number of plants investigated. The data reveals that the herbs sold in the urban area of Fangcheng are mainly collected from the Guangxi Fangcheng Golden Camellias national nature reserve, which is also confirmed by our interviewees. Because there is a certain distance from the wild habitat of the nature reserve, urban residents have less access to wild plants. Compared with the residents around the nature reserve, they may have less knowledge about medicinal plants.

The herbal trade chain, composed of villagers (herbal gatherers)–herbal vendors–therapists–urban residents, is also a chain of traditional knowledge dissemination. Among them, we also noticed that the herbal shop, as a platform for the concentration of wild genetic resources and traditional knowledge, also played a role in screening the knowledge of medicinal plants. In general, herbal medicine traders will actively choose herbs with large biomass (low collection cost and easy burden for customers) and relatively good efficacy to sell for a higher profit; over time, the types of herbs that urban residents can identify and name tend to be reduced. The effectiveness of treatment will also tend to be efficient. The physical distance may also be one of the reasons why the species amount of medicinal plants we recorded in urban areas are far fewer than those in villages.

However, in terms of plant usage and medicinal efficacy, the medicinal plants used in towns and villages tend to be very consistent, probably because (1) the people who use herbs in urban areas are older. There are also a large number of people who have moved in from the countryside, and they still maintain relatively primitive habits in daily health care. On the other hand, it may be because urban areas and villages are prone to similar diseases due to almost the same climatic environment. Hence, villages and urban residents consistently understand the climatic environment, illness, and herbal medicine usage.

In this case, large quantities of exotic traditional Chinese medicine (traditional Chinese medicine) are rarely recorded, and the reason may be: (1) the unique local traditional culture is the mainstream in the local area; (2) the trading chain of bulk traditional Chinese medicine is more complex. Often unable to use local materials locally, they need to be imported from other regions, which is not a priority for local small traders.

### Sustainability of medicinal plant collection

During the investigation, we also focused on whether the local collection and utilization of wild medicinal plants are sustainable and whether they will cause irreversible damage to the environment and resources of nature reserves. In fact, we find that local people follow some principles for collecting medicinal plants, which effectively ensures the sustainability of the wild environment. First, the areas where local people collect wild herbs are outside the core protected areas, and the relevant local regulations and policies also help to protect the core areas' ecological environment effectively. Secondly, the locals like to use fresh products, so the amount collected each time is small; otherwise, it is challenging to keep the plants fresh. According to the local people, they also have a custom in the collection process to “collect the big and keep small”. In drug use, the local people also have many combinations to replace the single prescription, so each plant's dosage is minimal.

Although whole plants are used the most in analyzing the medicinal parts, most are herbs. Herbs themselves tend to have large biomass, so the pressure of collection on the environment is less; the use of underground parts of plants is also quite extensive, accounting for 21%, which requires the local community to be vigilant and advocate to avoid it. Combined with the local people's collection and drug use habits, if only the herbal medicine around the core area is collected and used, it will not cause catastrophic losses to the resources in reserve. Therefore, commercial collection activities by medicinal collectors should be strictly prohibited in protected areas, and local residents may be allowed to collect a small number of medicinal materials needed in non-core protected areas. In addition, in protected areas and even in the buffer zone, we also see a lot of economic forest planting, such as star anise, cinnamon, Chinese fir, etc. Developing economic forest planting areas may significantly pressure biodiversity and the environment.

The medicinal plants recorded in this study include a total of 5 endangered species, ten near-threatened species, and 15 vulnerable species, accounting for 7.6% of all recorded medicinal species. Although the local people do not use many of these medicinal plants, they still need to arouse the vigilance of the management department or step up publicity and encourage residents to replace them with non-endangered plants.

### Suggestions

Based on our investigation and discussion, we would like to put forward some suggestions better to protect local wild plant resources and related traditional knowledge:

Blindly banning the collection, on the contrary, may lead to the demise of traditional knowledge. The publicity of protected plants should be increased to encourage local people to use other non-protected plants instead and try to choose non-underground parts in the choice of plants.

Increasing the corresponding scientific research, including the introduction, domestication, and large-scale planting of essential local herbs (higher RFC), can not only increase the income of the local people but also effectively protect the wild plant resources in reserve. Increase the exploration of the mechanism of medicinal plants and excavate their material basis to verify the scientific nature of these traditional utilization habits and lay a foundation for their development and utilization.

In addition, every older person is a treasure house of traditional knowledge, which can be recorded and protected as much as possible.

## Conclusions

The medicinal plants used in the villages near the Golden Camellia Nature Reserve are diverse, and the relevant traditional knowledge is relatively well preserved. The collection of medicinal materials by local people is sustainable. This study suggests that the local government should also protect relevant traditional knowledge in the decision-making process.

## Supplementary Information

**Additional file 1: Table S1.** The inventory of medicinal plants used around the Guangxi Fangcheng Golden Camellias national nature reserve.

## Data Availability

The author confirms that all data generated or analyzed during this study are included in this published article.

## Abbreviations

RFC: Relative frequency citation
ICF: Informant consensus factor
