# Supplementary material for: The medicinal plant used in the Guangxi Fangcheng Golden Camellias national nature reserve, a coastal region in southern China
Source: J Ethnobiol Ethnomed. 2023 Jul 27;19:32. doi: 10.1186/s13002-023-00605-4 (PMC10375688; doi:10.1186/s13002-023-00605-4)
Supplement: Supplementary file 1 — Additional file 1: Table S1. The inventory of medicinal plants used around the Guangxi Fangcheng Golden Camellias national nature reserve. [file 13002_2023_605_MOESM1_ESM.docx]

| Scientific name | Venacular name | Family name | Life form | Resource type | Medicinal part | Process methods | Medicinal effects | Endangered status | RFC in villages | RFC in markets | Voucher IDs |
| --- | --- | --- | --- | --- | --- | --- | --- | --- | --- | --- | --- |
| *Abrus pulchellus* subsp. *mollis* (Hance) Verdcourt |  | Fabaceae | liana | wild | leaf | mash and external apply | treat breas soures | LC | 0.29 | 0.27 | YY210910030 |
|  |  |  |  |  | whole plant | decoction for oral administration | treat inner fever, hepatitis |  |  |  |  |
| *Acorus calamus* var. *angustatus* Besser |  | Acoraceae | herb | wild | whole plant | decoction for medicinal bath | promote blood circulation and remove blood stasis, loosen tendons and activate collaterals, relieve fatigue，treat infantile common cold, runny nose | LC | 0.50 | 0.68 | YY210909032 |
|  |  |  |  |  |  | decoction for oral administration | treat cold |  |  |  |  |
| *Acronychia pedunculata* (Linnaeus) Miquel |  | Rutaceae | herb | wild | branches | decoction for medicinal bath | prevent infantile colds, dispel dampness | LC | 0.28 | 0.68 | HRC210907055 |
| *Actinidia eriantha* Benth. | huo li tuo | Actinidiaceae | liana | wild | root | decoction for oral administration | skin itching, treat rheumatism | LC | 0.04 | 0.00 | YY210908057 |
| *Adenia cardiophylla* (Masters) Engler |  | Passifloraceae | liana | wild | branches | decoction for medicinal bath | loosen tendons and activate collaterals, relieve fatigue | LC | 0.06 | 0.00 | YY201208025 |
|  |  |  |  |  | root bark | taken orally with washing water of rice | treat fish bone stuck on throat |  |  |  |  |
| *Adenia heterophylla* (Blume) Koorders | ruan qing teng | Passifloraceae | liana | wild | root | hold in the mouth | soften the bone stuck in the throat | LC | 0.06 | 0.00 | YY210120023 |
| *Adiantum flabellulatum* L. |  | Pteridaceae | herb | wild | whole plant | decoction for oral administration | treat diarrhea, dysentery, gastroenteritis and vomiting | LC | 0.13 | 0.00 | YY201208023 |
| *Adina pilulifera* (Lam.) Franch. ex Drake |  | Rubiaceae | shrub | wild | flower bud | decoction for oral administration | clear heat-toxin | LC | 0.17 | 0.32 | YY210121001 |
| *Adina rubella* Hance |  | Rubiaceae | tree | wild | branches | decoction for medicinal bath | calm children's nerve | LC | 0.09 | 0.00 | YY201209073 |
| *Aerva sanguinolenta* (Linnaeus) Blume |  | Amaranthaceae | herb | wild | whole plant | decoction for medicinal bath | promote blood circulation and remove blood stasis, remove dampness, relieve itching, relieve fatigue | LC | 0.19 | 0.55 | HRC210907033 |
| *Agave sisalana* Perrine ex Engelmann |  | Agavaceae | herb | cultivated | leaf | decoction for medicinal bath | treat measles | LC | 0.03 | 0.05 | HRC210907085 |
| *Agrimonia pilosa* Ledebour |  | Rosaceae | herb | wild | leaf | mash and external apply | stop bleeding | LC | 0.24 | 0.36 | YY201209029 |
| *Alchornea trewioides* (Benth.) Muell. Arg. |  | Euphorbiaceae | shrub | wild | branches | decoction for medicinal bath | remove dampness, promote blood circulation and remove blood stasis, relieve itching, relieve fatigue，prevent infantile colds, rash, treat dust allergy | LC | 0.38 | 0.82 | YY201209010 |
|  |  |  |  |  |  | mash and external apply | treat wound inflammation |  |  |  |  |
| *Alocasia cucullata* (Loureiro) G. Don in Sweet | lao hu yu | Araceae | herb | wild | stem | decoction for oral administration | treat a bad cold | LC | 0.21 | 0.23 | YY201208060 |
| *Alocasia odora* (Roxburgh) K. Koch | di shui guan yin | Araceae | herb | wild | stem | decoction with calclime for medicinal bath | treat smallpox | LC | 0.40 | 0.41 | YY210122015 |
| *Alpinia hainanensis* K. Schumann in Engler |  | Zingiberaceae | herb | cultivated | stem | decoction for oral administration | treat sore throat and diabetes | LC | 0.21 | 0.00 | YY210118049 |
| *Alpinia roxburghii* var. glabrior (Hand.-Mazz.) Saensouk | cao kou | Zingiberaceae | herb | wild | seed | decoction for oral administration | treat stomach disease | LC | 0.05 | 0.00 | YY210122007 |
| *Alsophila gigantea* Wall. ex Hook. | long gu feng | Cyatheaceae | herb | wild | stem | decoction for medicinal bath, medicinal wine for external application | treat rheumatism | LC | 0.19 | 0.09 | YY201209066 |
| *Alstonia scholaris* (Linnaeus) R. Brown |  | Apocynaceae | tree | cultivated | root | taken orally with washing water of rice | treat snake bites | LC | 0.03 | 0.00 | HRC210907063 |
|  |  |  |  |  | branches | decoction for medicinal bath | prevent infantile colds, remove dampness |  |  |  |  |
| *Amorphophallus variabilis* Blume |  | Araceae | herb | wild | tuber | medicinal soup | treat senile dementia | LC | 0.02 | 0.00 | YY201209057 |
| *Anisomeles indica* (Linnaeus) Kuntze |  | Lamiaceae | herb | wild | whole plant | orally taken directly, sleep on it | treat diarrhea | LC | 0.38 | 0.73 | HRC210906007 |
|  |  |  |  |  |  | decoction for medicinal bath | exorcise evil spirits, calm the nerve，remove prickly heat |  |  |  |  |
|  |  |  |  |  |  | sleep on it | treat Body swelling of newborn baby, infantile jaundice |  |  |  |  |
| Antidesma fordii Hemsley |  | Phyllanthaceae | tree | wild | root | mash and external apply | treat sore and furuncle | LC | 0.05 | 0.00 | YY201208017 |
| Aporosa octandra (Buch.-Ham. ex D.Don) Vickery |  | Phyllanthaceae | tree | wild | branches | decoction for medicinal bath | help would healing | LC | 0.45 | 0.86 | YY201209015 |
|  |  |  |  |  | leaf | mash and external apply bath | stop bleeding, help would healing |  |  |  |  |
| *Aralia chinensis* Linnaeus |  | Araliaceae | shrub | wild | root | decoction for oral administration | clear heat-toxin | LC | 0.03 | 0.00 | YY210118035 |
| *Aralia elata* (Miquel) Seemann | ying bu zhan | Araliaceae | tree | wild | whole plant | decoction for medicinal bath | treat fever | LC | 0.08 | 0.00 | YY210910018 |
| *Aralia finlaysoniana* (Wallich ex G. Don) Seemann | niao bu zhan | Araliaceae | shrub | wild | root | decoction for oral administration | treat diabetes, treat hepatitis B | LC | 0.19 | 0.00 | YY201209054 |
|  |  |  |  |  |  | mash and external apply | treat bone fracture |  |  |  |  |
| *Archidendron lucidum* (Bentham) I. C. Nielsen |  | Fabaceae | tree | wild | branches | decoction for washing hair | black hair | LC | 0.13 | 0.32 | YY210118038 |
| *Archidendron clypearia* (Jack) I. C. Nielsen | xi tou pi | Fabaceae | tree | wild | leaf | decoction for washing hair | black hair | LC | 0.29 | 0.27 | YY201208001 |
| *Ardisia crenata* Sims |  | Primulaceae | shrub | wild | branches | decoction for medicinal bath | treat sprain, promote blood circulation and remove blood stasis | LC | 0.17 | 0.09 | YY210121008 |
| *Ardisia fordii* Hemsley in F. B. Forbes & Hemsley |  | Primulaceae | shrub | wild | whole plant | mash and external apply | treat bruises, treat pulmonary tuberculosis | LC | 0.05 | 0.00 | YY210118015 |
| *Ardisia gigantifolia* Stapf |  | Primulaceae | shrub | wild | tuber, whole plant | mash and external apply, medicinal wine for oral administration, medicinal wine for external application | treat paralysis, treat rheumatism | LC | 0.44 | 0.23 | YY201209055 |
| *Ardisia hanceana* Mez in Engler | da luo san | Primulaceae | shrub | wild | branches | decoction for oral administration, medicinal wine for oral administration | treat rheumatism, relieve pain, treat bruises | LC | 0.25 | 0.36 | YY210910006 |
| *Ardisia polysticta* Miq. |  | Primulaceae | shrub | wild | leaf | mash and external apply | treat scald | LC | 0.11 | 0.00 | YY201209038 |
|  |  |  |  |  |  | medicinal wine for external application | treat rheumatism |  |  |  |  |
|  |  |  |  |  | root | mash and external apply | treat scald |  |  |  |  |
| *Ardisia quinquegona* var. *salicifolia* C.M.Hu & J.E.Vidal | xiao luo san | Primulaceae | shrub | wild | leaf | mash and external apply | treat osteoproliferation | LC | 0.07 | 0.00 | YY210119044 |
|  |  |  |  |  |  | decoction for medicinal bath | treat bruises |  |  |  |  |
|  |  |  |  |  | whole plant | decoction for medicinal bath | treat rheumatism |  |  |  |  |
| *Ardisia thyrsiflora* D. Don | tie luo san | Primulaceae | shrub | wild | branches | mash and external apply | treat bruises | LC | 0.17 | 0.14 | YY201209098 |
| *Ardisia villosa* Roxb. | tie luo san | Primulaceae | shrub | wild | whole plant | mash and external apply, decoction for medicinal bath | treat sprain, treat bruises | LC | 0.16 | 0.18 | YY210908021 |
| *Artemisia argyi* H. Leveille & Vaniot |  | Asteraceae | herb | wild | branches | decoction for medicinal bath | promote blood circulation and remove blood stasis, loosen tendons and activate collaterals, relieve fatigue, treat infantile common coldrunny nose | LC | 0.65 | 0.91 | YY210911006 |
| *Artemisia indica* Willdenow |  | Asteraceae | herb | wild | branches | decoction for medicinal bath | treat infantile colds and runny nose | LC | 0.51 | 0.86 | YY210909029 |
| *Asarum insigne* Diels |  | Aristolochiaceae | herb | wild | leaf | mash and external apply, medicinal wine for external application | relieve pain, anesthetic | VU | 0.06 | 0.00 | YY210121064 |
|  |  |  |  |  |  | medicinal wine for external application | treat bruises |  |  |  |  |
| *Asplenium antrophyoides* Christ |  | Aspleniaceae | herb | wild | leaf | mash and external apply | remove the scars on the head | LC | 0.09 | 0.14 | YY201209031 |
|  |  |  |  |  | whole plant | decoction for medicinal bath | treat infantile malnutrition |  |  |  |  |
| *Axonopus compressus* (Swartz) P. Beauvois |  | Poaceae | herb | wild | whole plant | decoction for medicinal bath | treat infantile colds and runny nose | LC | 0.27 | 0.27 | HRC210907007 |
| *Baeckea frutescens* Linnaeus |  | Myrtaceae | shrub | wild | whole plant, branches | decoction for medicinal bath | calm children's nerve, treat fever, colds, treat gynecological diseases, remove dampness, treat dust allergy | LC | 0.47 | 0.68 | YY210119005 |
| *Bambusa chungii* McClure |  | Poaceae | herb | cultivated | stem | heat it for the bamboo juice and taken orally | clear heat-toxin | LC | 0.33 | 0.00 | YY201208054 |
| *Bauhinia championii* var. *apertilobata* (Merr. & F.P.Metcalf) M.Hiroe |  | Fabaceae | liana | wild | branches | decoction for oral administration, decoction for medicinal bath | treat rheumatism, treat measles | NT | 0.10 | 0.09 | YY210121028 |
| *Begonia hemsleyana* J. D. Hooker |  | Begoniaceae | herb | wild | whole plant | mash and external apply | treat sore and furuncle | LC | 0.03 | 0.00 | YY210121056 |
| *Begonia rhynchocarpa* Y. M. Shui & W. H. Chen | da ban ye lian | Begoniaceae | herb | wild | whole plant | decoction for oral administration | treat rheumatism | NT | 0.06 | 0.09 | YY201209050 |
| *Berchemia polyphylla* Wall. | mao shi guo | Rhamnaceae | shrub | wild | root | decoction for oral administration | treat hepatitis and pneumonia | LC | 0.03 | 0.00 | YY201209012 |
| *Bidens alba* (L.) DC. |  | Asteraceae | herb | wild | root | decoction for medicinal bath | clear heat-toxin | LC | 0.38 | 0.27 | HRC210906006 |
| *Bischofia polycarpa* (H. Leveille) Airy Shaw |  | Phyllanthaceae | tree | wild | branches | decoction for medicinal bath | relieve fatigue, relieve muscle pain, promote blood circulation and remove blood stasis, treat rheumatism, treat headache, help postpartum recovery | LC | 0.22 | 0.23 | HRC210907044 |
| *Blastus cochinchinensis* Loureiro |  | Melastomataceae | shrub | wild | branches | decoction for medicinal bath | relieve itching, calm the nerve, calm children‘s nerve | LC | 0.32 | 0.55 | YY210121049 |
| *Blumea aromatica* Candolle | huo you cai | Asteraceae | herb | wild | branches | decoction for oral administration | treat hepatosplenomegaly | LC | 0.09 | 0.00 | YY210119076 |
| *Blumea balsamifera* (Linnaeus) Candolle |  | Asteraceae | herb | wild | branches | decoction for medicinal bath | treat rheumatism, promote blood circulation and remove blood stasis, relieve fatigue, relieve muscle pain, treat headache, treat infantile common coldrunny nose, relieve fatigue, help postpartum recovery | LC | 0.42 | 0.95 | YY210601010 |
| *Blumea megacephala* (Randeria) C. C. Chang & Y. Q. Tseng in Y. Ling | jiu li ming | Asteraceae | liana | wild | branches | decoction for medicinal bath | treat pustule | LC | 0.36 | 0.82 | YY210119010 |
| *Blumea riparia* Candolle |  | Asteraceae | liana | wild | branches | decoction for medicinal bath | treat infantile jaundice | LC | 0.26 | 0.59 | HRC210907068 |
|  |  |  |  |  |  | decoction for oral administration | treat high fever |  |  |  |  |
| *Boehmeria penduliflora* Weddell ex D. G. Long |  | Urticaceae | shrub | wild | branches | decoction for medicinal bath | treat knife wounds and inflamed wounds | LC | 0.02 | 0.00 | YY210121030 |
| *Breynia fruticosa* (Linnaeus) Muller Argoviensis in A. Candolle |  | Phyllanthaceae | shrub | wild | branches | decoction for oral administration | treat diarrhea, vomiting, snake bites | LC | 0.17 | 0.32 | YY210531002 |
|  |  |  |  |  |  | decoction for medicinal bath | treat gynecological diseases, treat infantile common coldtreat fever |  |  |  |  |
| *Breynia rostrata* Merrill |  | Phyllanthaceae | shrub | wild | leaf | decoction for oral administration | treat rheumatism, stop pain, treat snake bites | LC | 0.16 | 0.27 | YY201209011 |
| *Broussonetia kaempferi* Sieb. |  | Moraceae | shrub | wild | leaf | mash and external apply | treat sore and furuncle, treat tetanus | LC | 0.29 | 0.27 | YY210120024 |
| *Bulbophyllum kwangtungense* Schlechter |  | Orchidaceae | herb | wild | whole plant | decoction for oral administration | Clear heat and remove phlegm | LC | 0.16 | 0.23 | YY201209046 |
| *Callerya cinerea* (Bentham) Schot |  | Fabaceae | liana | wild | branches | decoction for medicinal bath | promote blood circulation and remove blood stasis | LC | 0.08 | 0.00 | YY210122020 |
| *Callerya speciosa* (Champ. ex Benth.) Schot |  | Fabaceae | liana | wild | root | medicinal wine for oral administration | nourish the body, treat rheumatism, tonify Yang | LC | 0.41 | 0.41 | YY201208053 |
| *Callicarpa* formosana Rolfe |  | Lamiaceae | shrub | wild | root | mash and external apply | treat sore and furuncle | LC | 0.16 | 0.14 | YY201209024 |
|  |  |  |  |  |  | decoction for oral administration | treat dysentery |  |  |  |  |
| *Callicarpa kwangtungensis* Chun | jiang feng cha | Lamiaceae | shrub | wild | branches | decoction for medicinal bath | treat infantile fever, nfantile jaundice, calm the nerve | LC | 0.19 | 0.14 | YY210910031 |
| *Calophyllum membranaceum* Gardner & Champion |  | Calophyllaceae | tree | wild | leaf | mash and external apply | treat sore and furuncle | VU | 0.27 | 0.14 | YY210121040 |
|  |  |  |  |  |  | decoction for oral administration | treat rheumatism |  |  |  |  |
|  |  |  |  |  | root | medicinal wine for oral administration | treat rheumatism |  |  |  |  |
| *Camellia drupifera* Loureiro |  | Theaceae | tree | wild | branches | decoction for medicinal bath | remove dampness, promote blood circulation and remove blood stasis, loosen tendons and activate collaterals, relieve fatigue, prevent infantile colds, runny nose | LC | 0.57 | 0.82 | HRC210907056 |
| *Camellia sinensis* (Linnaeus) Kuntze |  | Theaceae | shrub | cultivated | branches | decoction for medicinal bath | dispel dampness and treat dust allergy | DD | 0.25 | 0.27 | YY210911030 |
| *Canavalia gladiata* (Jacquin) Candolle | song jin teng | Fabaceae | herb | wild | fruit | roast and external apply | treat mumps | LC | 0.04 | 0.00 | YY210118002 |
| *Cassytha filiformis* Linnaeus |  | Lauraceae | herb | wild | whole plant | decoction for medicinal bath | treat rheumatism, promote blood circulation and remove blood stasis, relieve itching, relieve fatigue, infantile common cold，treat coldtreat fever | LC | 0.28 | 0.64 | HRC210907009 |
|  |  |  |  |  |  | mash and exteral apply | treat sore and furuncle, haemorrhoids, acute mastitis |  |  |  |  |
| *Catunaregam spinosa* (Thunberg) Tirvengadum |  | Rubiaceae | shrub | wild | branches | decoction for medicinal bath | treat hepatitis and pneumonia | LC | 0.06 | 0.00 | YY210531020 |
| *Celosia argentea* Linnaeus |  | Amaranthaceae | herb | wild | seed | decoction for oral administration | clear heat in livers | LC | 0.20 | 0.27 | YY210122006 |
| *Centella asiatica* (Linnaeus) Urban in Martius |  | Apiaceae | herb | wild | whole plant | mash and external apply | treat pimple | LC | 0.63 | 0.91 | HRC210906008 |
|  |  |  |  |  |  | decoction for oral administration | clear heat-toxin, lymphatic nodules |  |  |  |  |
| *Cheniella glauca* (Benth.) R. Clark & Mackinder | da zhong yang ti jia | Fabaceae | liana | wild | branches | mash and external apply, decoction for medicinal bath | treat sprain, treat bruises | LC | 0.18 | 0.23 | YY210908023 |
| *Chromolaena corymbosa* (Aubl.) R.M.King & H.Rob. |  | Asteraceae | herb | wild | leaf | mash and external apply on the palm and foot arch | treat fever, treat beriberi | LC | 0.27 | 0.00 | HRC210906009 |
| *Chromolaena odorata* (Linnaeus) R. M. King & H. Robinson |  | Asteraceae | herb | wild | leaf | mash and external apply | treat knife wound, stop bleeding | LC | 0.31 | 0.00 | YY210121063 |
|  |  |  |  |  | whole plant | decoction for medicinal bath | treat cold, remove dampness |  |  |  |  |
|  |  |  |  |  |  | decoction for oral administration | sore throat |  |  |  |  |
| *Cinnamomum parthenoxylon* (Jack) Meisner in A. Candolle | jiang mu | Lauraceae | tree | wild | camphor | medicinal soup | treat dementia | LC | 0.08 | 0.00 | YY210908003 |
| *Cissus hexangularis* Thorel ex Planchon in A. Candolle & C. Candolle | da luo shu | Vitaceae | liana | wild | root | decoction for medicinal bath | treat rheumatism | LC | 0.21 | 0.41 | YY210910040 |
|  |  |  |  |  | whole plant | therapeutic diet | treat lumbago, treat bruises |  |  |  |  |
| *Citrus maxima* (Burman) Merrill |  | Rutaceae | tree | cultivated | branches | decoction for medicinal bath | promote blood circulation and remove blood stasis, relieve itching, relieve fatigue, treat infantile common coldrunny nose | LC | 0.52 | 0.82 | HRC210907020 |
| *Clausena anisata* (Willd.) Hook.f. | shan huang pi | Rutaceae | tree | wild | leaf | decoction for medicinal bath | treat bruises, infantile jaundice，root | LC | 0.33 | 0.73 | YY201209043 |
|  |  |  |  |  | root | decoction for oral administration | treat stomachache, liver pain, abdominal pain |  |  |  |  |
| *Clausena lansium* (Loureiro) Skeels |  | Rutaceae | tree | cultivated | branches | decoction for medicinal bath | promote blood circulation and remove blood stasis, relieve itching, relieve fatigue, calm children's nerve, treat fever | LC | 0.57 | 0.86 | HRC210907021 |
| *Cleidion brevipetiolatum* Pax & K. Hoffmann in Engler | hu li luan | Euphorbiaceae | tree | wild | root | decoction for medicinal bath | treat rheumatism, reliev iching | LC | 0.13 | 0.00 | YY210118027 |
| *Clematis armandii* Franchet | lao hu xu | Ranunculaceae | liana | wild | whole plant | decoction for oral administration | treat rheumatism | LC | 0.09 | 0.00 | YY210118025 |
| *Clematis grandidentata* (Rehder & E. H. Wilson) W. T. Wang | lao hu xu | Ranunculaceae | liana | wild | root | medicinal wine for oral administration | treat rheumatism | LC | 0.06 | 0.00 | YY210119027 |
| *Clerodendrum bungei* Steudel |  | Lamiaceae | shrub | wild | leaf | mash and external apply | relieve itching, treat decay | LC | 0.26 | 0.73 | YY210118004 |
| *Clerodendrum cyrtophyllum* Turczaninow |  | Lamiaceae | shrub | wild | branches | decoction for medicinal bath | treat rheumatism, promote blood circulation and remove blood stasis, relieve itching, relieve fatigue, treat infantile common coldtreat fever, runny nose, treat dust allergy | LC | 0.68 | 1.00 | YY201208026 |
| *Codonopsis javanica* Hook.f. & Thomson |  | Campanulaceae | liana | wild | root | medicinal soup | nourish the body | LC | 0.26 | 0.23 | YY201209075 |
| *Clinacanthus nutans* (N. L. Burman) Lindau | she qing | Acanthaceae | herb | cultivated | stem, leaf | decoction for oral administration | treat pulmonary edema | LC | 0.06 | 0.00 | YY201209087 |
| *Codonopsis pilosula* (Franchet) Nannfeldt |  | Campanulaceae | liana | wild | tuberous root | medicinal soup | nourish the body | LC | 0.28 | 0.27 | YY210121047 |
| *Commelina diffusa* N. L. Burman |  | Commelinaceae | herb | wild | whole plant | decoction for oral administration | clear liver and improve vision, treat hepatitis B | LC | 0.41 | 0.59 | YY210120006 |
| *Coptis chinensis* Franch. |  | Ranunculaceae | herb | cultivated | root | hold in the mouth | treat oral inflammation | VU | 0.02 | 0.00 | HRC210907094 |
| *Corchorus capsularis* Linnaeus |  | Malvaceae | herb | wild | branches | decoction for medicinal bath | treat infantile measles | LC | 0.05 | 0.00 | HRC210907084 |
| *Cordia dichotoma* G. Forster |  | Boraginaceae | tree | wild | bark | decoction for medicinal bath | relieve itching | LC | 0.02 | 0.00 | YY210118040 |
| *Cordyline fruticosa* (Linnaeus) A. Chevalier | tie shu | Asparagaceae | shrub | wild | branches | decoction for oral administration | cool the blood | LC | 0.03 | 0.00 | YY210119084 |
| *Costus tonkinensis* Gagnepain |  | Costaceae | herb | wild | tuber | decoction for oral administration | Relieve heat | LC | 0.02 | 0.00 | YY201209090 |
| *Cratoxylum cochinchinense* (Loureiro) Blume |  | Hypericaceae | tree | wild | bark | mash and external apply | treat snake bites | LC | 0.48 | 0.91 | HRC210907012 |
|  |  |  |  |  | branches, leaf | decoction for medicinal bath | remove dampnessrelieve itching, treat coldtreat fever, infantile measles |  |  |  |  |
| *Crinum asiaticum* var. *sinicum* (Roxburgh ex Herbert) Baker | dong suan tou | Amaryllidaceae | herb | cultivated | leaf | roast and external apply | treat rheumatism | LC | 0.03 | 0.05 | YY201209076 |
| *Crotalaria assamica* Bentham | ding xin rong | Fabaceae | herb | wild | tuber, leaf | therapeutic diet | treat heart diseases | LC | 0.22 | 0.18 | YY210120001 |
| *Crotalaria sessiliflora* L. |  | Fabaceae | herb | wild | tuber | stew with chicken | nourish the body | LC | 0.04 | 0.00 | YY210118047 |
| *Croton tiglium* Linnaeus |  | Euphorbiaceae | shrub | wild | fruit | orally taken directly | treat constipation | LC | 0.08 | 0.41 | YY210118045 |
|  |  |  |  |  | root | decoction for medicinal bath | treat rheumatism |  |  |  |  |
| *Cunninghamia lanceolata* (Lambert) Hooker |  | Cupressaceae | tree | wild | branches | decoction for medicinal bath | remove dampness, treat infantile common cold, runny nose, infantile jaundice | LC | 0.29 | 0.32 | HRC210907066 |
| *Curcuma longa* Linnaeus | huang jiang | Zingiberaceae | herb | cultivated | tuber | decoction for oral administration | loosen tendons and activate collaterals | LC | 0.36 | 0.27 | YY210122008 |
| *Cuscuta japonica* Choisy |  | Convolvulaceae | herb | wild | vine | decoction for medicinal bath | treat bruises, treat bone fracture, treat sprain | LC | 0.21 | 0.09 | YY201208041 |
| *Cyclea hypoglauca* (Schauer) Diels in Engler |  | Menispermaceae | liana | wild | stem | medicinal wine for oral administration | treat rheumatism | LC | 0.16 | 0.27 | YY201208034 |
| *Cymbopogon citratus* (Candolle) Stapf |  | Poaceae | herb | wild | whole plant | decoction for medicinal bath | promote blood circulation and remove blood stasis, loosen tendons and activate collaterals, relieve fatigue, treat infantile common cold | LC | 0.38 | 0.82 | YY201209071 |
| *Dalbergia hancei* Bentham | pen dong teng | Fabaceae | liana | wild | branches and leaf | decoction for washing hair | black hair；root | LC | 0.17 | 0.18 | YY201209020 |
|  |  |  |  |  | root or leaf | mash and external apply | stop bleeding, help wound healing, treat traumatic injury |  |  |  |  |
| *Daphniphyllum calycinum* Bentham |  | Daphniphyllaceae | shrub | wild | branches, root | decoction for medicinal bath, mix with honey for oral administration, medicinal wine for oral administration | treat rheumatism | LC | 0.38 | 0.36 | YY201209028 |
| *Davallia formosana* Hayata |  | Davalliaceae | herb | wild | stem | mash and external apply | treat bone fracture, treat rheumatism | LC | 0.32 | 0.36 | YY201209058 |
| *Decaneuropsis cumingiana* (Benth.) H.Rob. & Skvarla | guo shan long | Asteraceae | liana | wild | branches | decoction for medicinal bath | reduce swelling | LC | 0.27 | 0.27 | YY201208033 |
|  |  |  |  |  | root | medicinal wine for oral administration, decoction for oral administration, decoction for medicinal bath | treat rheumatism, treat rheumatism, treat scald, treat skin diseases, treat sore throat, diarrhea |  |  |  |  |
| *Decaspermum gracilentum* (Hance) Merrill & L. M. Perry | song bai mu | Myrtaceae | tree | wild | branches | decoction for medicinal bath | relieve fatigue | LC | 0.27 | 0.27 | YY210118022 |
| *Dendrobium devonianum* Paxton |  | Orchidaceae | herb | wild | whole plant | decoction for oral administration | clear heat and remove phlegm | EN | 0.03 | 0.00 | YY201209047 |
| *Dendrolobium triangulare* (Retzius) Schindler |  | Fabaceae | shrub | wild | branches | decoction for medicinal bath | relieve itching and fatigue | LC | 0.10 | 0.00 | HRC210906011 |
| *Derris fordii* Oliver | tie teng | Fabaceae | shrub | wild | root | decoction for medicinal bath | treat bruises, promote blood circulation and remove blood stasis | LC | 0.06 | 0.00 | YY210118013 |
| *Derris trifoliata* Loureiro |  | Fabaceae | liana | wild | stem | decoction for medicinal bath, medicinal wine for external application | treat rheumatism, treat bruises | LC | 0.11 | 0.00 | YY210118041 |
| *Desmos chinensis* Loureiro | ying zhua feng | Annonaceae | shrub | wild | branches | decoction for medicinal bath | loosen tendons and activate collaterals,treat rheumatism | LC | 0.38 | 0.82 | YY210118029 |
| *Dicliptera chinensis* (Linnaeus) Jussieu |  | Acanthaceae | herb | wild | whole plant | decoction for medicinal bath, decoction for oral administration | treat infantile jaundice, clear inner heat, relieve pain, reduce inflammation | LC | 0.19 | 0.23 | HRC210907071 |
| *Dicliptera tinctoria* Kostel. |  | Acanthaceae | herb | wild | branches | decoction for medicinal bath | treat infantile jaundice | LC | 0.11 | 0.41 | HRC210907069 |
| *Dicranopteris pedata* (Houtt.) Nakaike |  | Gleicheniaceae | herb | wild | whole plant | decoction for medicinal bath | treat skin allergy | LC | 0.05 | 0.00 | YY210910022 |
| *Dicranopteris splendida* (Hand.-Mazz.) Tagawa | tie mang ji | Gleicheniaceae | herb | wild | tender shoot | chewing until it is sweet and then cover on the wounded part | treat centipede poisoning | LC | 0.03 | 0.00 | YY210119017 |
| *Dimocarpus longan* Loureiro |  | Sapindaceae | tree | cultivated | branches | decoction for medicinal bath | prevent infantile colds and dispel dampness | LC | 0.17 | 0.27 | HRC210907052 |
| *Dioscorea pentaphylla* Linnaeus |  | Dioscoreaceae | liana | wild | leaf | medicinal wine for oral administration | treat bruises, treat bone fracture | LC | 0.16 | 0.27 | YY201208006 |
|  |  |  |  |  |  | decoction for oral administration | treat hepatitis |  |  |  |  |
|  |  |  |  |  | tuberous root, leaf | medicinal wine for external application | medicinal wine for external applicationtreat rheumatism |  |  |  |  |
| *Dischidia chinensis* Champion ex Bentham |  | Apocynaceae | liana | wild | whole plant | mash and external apply, decoction for medicinal bath | treat herpes zoster | LC | 0.09 | 0.00 | HRC210906003 |
| *Dracaena reflexa* Lam. | shan zhu mu | Asparagaceae | herb | cultivated | leaf | decoction for medicinal bath | treat syphilis | LC | 0.08 | 0.00 | YY201209053 |
|  |  |  |  |  | whole plant | decoction for oral administration | promote blood circulation and remove blood stasis, treat paralysis |  |  |  |  |
| *Drynaria coronans* J.Sm. |  | Polypodiaceae | herb | wild | tuber | decoction for medicinal bath | treat rheumatism, treat tinea | LC | 0.29 | 0.27 | YY210121050 |
| *Drynaria roosii* Nakaike | hou zi xie | Polypodiaceae | herb | wild | stem | decoction for oral administration | clear heat-toxin, treat cough | LC | 0.33 | 0.73 | YY210120013 |
| *Duchesnea indica* (Andrews) Focke in Engler & Prantl |  | Rosaceae | herb | wild | whole plant | mash and make tea | detoxification | LC | 0.02 | 0.00 | YY210121033 |
| *Eclipta prostrata* (Linnaeus) Linnaeus |  | Asteraceae | herb | wild | whole plant | mash and external apply, decoction for oral administration | treat sore and furuncle | LC | 0.21 | 0.14 | YY210121039 |
| *Ehretia acuminata* R. Brown |  | Boraginaceae | tree | wild | branches | decoction for medicinal bath | relieve fatigue, promote blood circulation and remove blood stasis | NT | 0.08 | 0.00 | YY210909003 |
| *Elephantopus scaber* Linnaeus |  | Asteraceae | herb | wild | whole plant | decoction for medicinal bath | calm children’s nerve, treat fever | LC | 0.65 | 0.82 | YY210531007 |
| *Eleusine indica* (Linnaeus) Gaertner |  | Poaceae | herb | wild | whole plant | decoction for oral administration | treat colds | LC | 0.03 | 0.00 | HRC210906001 |
| *Eleutherococcus trifoliatus* (Linnaeus) S. Y. Hu |  | Araliaceae | shrub | cultivated | stem | decoction for oral administration | treat hepatitis, pulmonary edema and hypertension | LC | 0.13 | 0.23 | YY210118042 |
| *Embelia laeta* (Linnaeus) Mez in Engler |  | Primulaceae | liana | wild | root bark | steam with pork liver | treat infantile malnutrition, treat coldtreat cough | LC | 0.33 | 0.50 | YY201208050 |
| *Embelia ribes* N. L. Burman | niu wei wu | Primulaceae | liana | wild | root | decoction for oral administration | treat cough and clear lungs | LC | 0.06 | 0.00 | YY210118051 |
| *Emilia sonchifolia* (L.) DC. |  | Asteraceae | herb | wild | leaf | mash and external apply | treat sore and furuncle | LC | 0.22 | 0.41 | YY210121062 |
|  |  |  |  |  | whole plant | decoction for oral administration, mash and external apply | clear heat-toxin |  |  |  |  |
| *Engelhardia roxburghiana* Wallich | huang gu mu | Juglandaceae | tree | wild | leaf | decoction for medicinal bath | relieve itching | LC | 0.36 | 0.50 | YY210118036 |
| *Entada phaseoloides* (Linnaeus) Merrill |  | Fabaceae | liana | wild | stem | medicinal wine for oral administration, medicinal wine for external application | treat rheumatism | EN | 0.08 | 0.09 | YY201209056 |
| *Epipremnum aureum* (Linden et André) G. S. Bunting | ya jiao teng | Araceae | liana | wild | whole plant | decoction for medicinal bath | treat rheumatism | LC | 0.08 | 0.00 | YY201209030 |
| *Eranthemum pulchellum* Andrews |  | Acanthaceae | shrub | wild | root | decoction for oral administration | clear inner heat | LC | 0.01 | 0.00 | YY210122012 |
| *Eria scabrilinguis* Lindl. |  | Orchidaceae | herb | wild | whole plant | decoction for oral administration | treat cough and clear lungs | LC | 0.18 | 0.00 | YY210120022 |
| *Eriocaulon buergerianum* Kornicke in Miquel | gu jing cao | Eriocaulaceae | herb | wild | whole plant | decoction for medicinal bath, decoction for oral administration | treat cataract | LC | 0.09 | 0.23 | YY210119022 |
| *Erycibe obtusifolia* Bentham |  | Convolvulaceae | liana | wild | whole plant | decoction for medicinal bath | treat rheumatism, promote blood circulation and remove blood stasis, treat paralysis | VU | 0.08 | 0.00 | YY201209096 |
| *Erythropalum scandens* Blume | chou pi teng | Olacaceae | liana | wild | stem | decoction for oral administration | treat diabetes | LC | 0.13 | 0.14 | YY210908056 |
| *Eucalyptus robusta* Smith |  | Myrtaceae | tree | cultivated | branches | decoction for medicinal bath | dispel dampness and treat dust allergy | LC | 0.44 | 0.82 | YY210911038 |
| *Eupatorium fortunei* Turczaninow |  | Asteraceae | herb | wild | branches | decoction for oral administration | treat rheumatism, treat bruises | LC | 0.40 | 0.64 | YY201208043 |
|  |  |  |  |  | whole plant | decoction for medicinal bath | treat constipation |  |  |  |  |
| *Euphorbia hirta* Linnaeus |  | Euphorbiaceae | herb | wild | whole plant | decoction for medicinal bath | dispel dampness and treat dust allergy | LC | 0.31 | 0.14 | YY210911035 |
| *Euphorbia royleana* Boissier in A. Candolle |  | Euphorbiaceae | shrub | wild | stem | mash and external apply | treat osteoproliferation | LC | 0.07 | 0.00 | YY210120003 |
|  |  |  |  |  |  | therapeutic diet | treat infantile malnutrition |  |  |  |  |
| *Fibraurea recisa* Pierre | xing teng | Menispermaceae | liana | wild | stem | decoction for oral administration | treat diabetes and hepatitis | LC | 0.05 | 0.00 | YY210118021 |
| *Ficus abelii* Miquel | shui rong shu | Moraceae | shrub | wild | leaf | decoction for medicinal bath | calm the nerve of children | LC | 0.10 | 0.09 | YY210908040 |
| *Ficus auriculata* Loureiro |  | Moraceae | tree | wild | fruit | therapeutic diet | promote lactation | LC | 0.06 | 0.00 | YY201209044 |
| *Ficus hispida* Linnaeus f. |  | Moraceae | tree | wild | branches | decoction for medicinal bath | treat rheumatism | LC | 0.06 | 0.00 | YY210601003 |
| *Ficus simplicissima* Lour. | xiao wu zhi niu nai | Moraceae | shrub | wild | root | medicinal soup, decoction for oral administration | treat hepatitis B, tonify the kidney, nourish the body | LC | 0.48 | 0.59 | YY201208002 |
| *Ficus subpisocarpa* Gagnepain | bo luo rong | Moraceae | tree | wild | branches | medicinal tea | treat bruises, promote blood circulation and remove blood stasis | LC | 0.07 | 0.00 | YY210120007 |
| *Fimbristylis ovata* (N. L. Burman) J. Kern | shui zhen cao | Cyperaceae | herb | wild | whole plant | decoction for oral administration | treat hypertension | LC | 0.03 | 0.00 | YY210119021 |
| *Firmiana pulcherrima* H. H. Hsue |  | Sterculiaceae | tree | wild | stem | mash and cover nose | treat rhinitis | EN | 0.03 | 0.00 | YY210910008 |
| *Fissistigma chloroneurum* (Handel-Mazzetti) Tsiang |  | Annonaceae | shrub | wild | whole plant | decoction for medicinal bath | treat rheumatism | LC | 0.17 | 0.14 | YY210121019 |
| *Flemingia macrophylla* (Willdenow) Prain |  | Fabaceae | shrub | wild | whole plant | decoction for medicinal bath | treat rheumatism, treat fatigue, nourish the body,calm children‘s nerve | LC | 0.27 | 0.23 | YY210118031 |
| *Flemingia prostrata* Roxburgh | san zhang ye | Fabaceae | shrub | wild | root | decoction for oral administration | treat pneumonia and bronchitis | LC | 0.03 | 0.00 | YY210119009 |
| *Flueggea virosa* (Roxburgh ex Willdenow) Voigt | qian chui da | Phyllanthaceae | shrub | wild | branches | external apply | treat hemorrhoids, treat rheumatism | LC | 0.21 | 0.36 | YY201209039 |
| *Garcinia oblongifolia* Champion ex Bentham | luo meng | Guttifera | tree | wild | pericarp | taken orally directly | treat sore throat | LC | 0.06 | 0.00 | YY210908001 |
| *Gardenia jasminoides* J. Ellis |  | Rubiaceae | shrub | wild | fruit | decoction for oral administration | clear heat-toxin | LC | 0.19 | 0.00 | YY210120008 |
|  |  |  |  |  | root | therapeutic diet | treat hepatitis, toothachein |  |  |  |  |
| *Gladiolus × gandavensis* Van Houtte |  | Iridaceae | herb | cultivated | whole plant | decoction for medicinal bath | promote blood circulation and remove blood stasis, remove dampness, relieve itching, relieve fatigue, prevent infantile colds | LC | 0.09 | 0.14 | HRC210907026 |
| *Gnetum montanum* Markgraf | hong teng | Gnetaceae | liana | wild | stem, branches | medicinal wine for oral administration, decoction for medicinal bath | treat rheumatism, treat measles, calm children‘s nerve | LC | 0.31 | 0.00 | YY210119055 |
| *Gomphandra tetrandra* (Wallich) Sleumer | shan luo bo | Stemonuraceae | tree | wild | root, leaf | decoction for oral administration | clear heat-toxin, dispel the effects of alcohol, cool the blood, treat internal injury, treat hepatitis B | LC | 0.17 | 0.00 | YY210908027 |
| *Grangea maderaspatana* (Linnaeus) Poiret | tian ji huang | Asteraceae | herb | wild | whole plant | decoction for oral administration | treat hepatitis B | LC | 0.61 | 1.00 | YY210119070 |
| *Grona heterocarpa* var. *strigosa* (Meeuwen) H.Ohashi & K.Ohashi | jia hua sheng | Fabaceae | shrub | wild | branches | decoction for medicinal bath | remove dampness, promote blood circulation and remove blood stasis, relieve itching, relieve fatigue | LC | 0.22 | 0.36 | YY210910021 |
|  |  |  |  |  |  | mash and external apply | treat haemorrhoids |  |  |  |  |
| *Grona styracifolia* (Osbeck) H.Ohashi & K.Ohashi |  | Fabaceae | herb | wild | branches | decoction for medicinal bath | treat infantile jaundice | LC | 0.20 | 0.55 | HRC210907074 |
| *Gymnema sylvestre* (Retzius) Schultes in Roemer & Schultes | hua dong zhong | Apocynaceae | liana | wild | whole plant | mash and external apply | treat rheumatism | LC | 0.17 | 0.09 | YY210120018 |
| *Gynura japonica* (Thunberg) Juel | shi lan cai | Asteraceae | herb | wild | whole plant | decoction for medicinal bath | treat bruises | LC | 0.02 | 0.00 | YY201208058 |
| *Habenaria rhodocheila* Hance | shen long cao | Orchidaceae | herb | wild | tuberous root | decoction for oral administration | relieve the kidney pain, tonify the kidney | LC | 0.03 | 0.00 | YY210908034 |
| *Hedyotis auricularia* L. |  | Rubiaceae | herb | wild | whole plant | decoction for medicinal bath | treat infantile jaundice | LC | 0.17 | 0.14 | HRC210907072 |
| *Hedyotis effusa* Hance | long gou gan | Rubiaceae | herb | wild | whole plant | decoction for oral administration | clear heat-toxin | LC | 0.50 | 0.73 | YY210120032 |
| *Heliciopsis lobata* (Merrill) Sleumer | dong zhang feng | Proteaceae | tree | wild | root, leaf | decoction for medicinal bath | treat rheumatism, treat bruises, help postpartum recovery | LC | 0.08 | 0.00 | YY210119031 |
| *Hellenia speciosa* (J.Koenig) Govaerts | a po jie | Costaceae | herb | wild | tuber | decoction for oral administration | clear heat-toxin | LC | 0.20 | 0.00 | YY210908004 |
| *Smilax bockii* Warb. ex Diels | jin gang teng | Smilacaceae | shrub | wild | tender shoot | mash and external apply | treat sore and furuncle | LC | 0.08 | 0.00 | YY201208010 |
|  |  |  |  |  | tuberous root | medicinal wine for oral administration | medicinal wine for oral administration: nourish the boday |  |  |  |  |
| *Heynea velutina* F. C. How & T. C. Chen | hei xiao | Meliaceae | shrub | wild | tuberous root | mash and external apply | treat bone fracture | LC | 0.01 | 0.00 | YY210118024 |
| *Homalomena occulta* (Loureiro) Schott in Schott & Endlicher |  | Araceae | herb | cultivated | stem | roast and external apply | treat rheumatism | LC | 0.25 | 0.27 | YY201209084 |
| *Homonoia riparia* Lour. |  | Euphorbiaceae | shrub | wild | root | decoction for oral administration | treat toothache | LC | 0.02 | 0.00 | YY210118011 |
| *Houttuynia cordata* Thunberg |  | Saururaceae | herb | wild | whole plant | decoction for medicinal bath | calm the mind | LC | 0.42 | 1.00 | YY210531016 |
| *Hoya fusca* Wallich | tie niu shan | Apocynaceae | shrub | wild | whole plant | decoction for medicinal bath | treat bruises | LC | 0.06 | 0.14 | YY210118008 |
| *Hoya fungii* Merr. | da shi long | Apocynaceae | liana | wild | branches | mash and external apply | treat bruises, treat bone fracture, promote blood circulation and remove blood stasis | LC | 0.05 | 0.00 | YY201208066 |
| *Hoya pottsii* Traill |  | Apocynaceae | shrub | wild | branches | decoction for medicinal bath | prevent infantile colds, remove dampness | LC | 0.06 | 0.00 | HRC210907065 |
|  |  |  |  |  | leaf | therapeutic diet | treat infantile malnutrition |  |  |  |  |
| *Huperzia carinata* (Desv. ex Poir.) Trevis. | ma wei diao | Lycopodiaceae | herb | wild | whole plant | medicinal wine for oral administration, medicinal wine for external application | treat rheumatism | VU | 0.17 | 0.14 | YY201209034 |
| *Huperzia phlegmaria* (L.) Rothm. | shang shu lian | Lycopodiaceae | herb | wild | whole plant | decoction for oral administration | treat pulmonary edema, remove prickly heat | VU | 0.09 | 0.09 | YY210118007 |
| *Hydrangea febrifuga* (Lour.) Y.De Smet & C.Granados | feng bi ma | Hydrangeaceae | shrub | wild | branches | mash and external apply | treat skin allergy | LC | 0.32 | 0.73 | YY210910009 |
| *Hypericum japonicum* Thunberg in Murray |  | Hypericaceae | herb | wild | whole plant | decoction for medicinal bath | treat infantile jaundice | LC | 0.36 | 1.00 | HRC210907073 |
| *Ilex hainanensis* Merrill |  | Aquifoliaceae | tree | wild | branches | decoction for medicinal bath | promote blood circulation and remove blood stasis, relieve itching, relieve fatigue | LC | 0.25 | 0.27 | YY201208036 |
| *Ilex rotunda Thunberg* in Murray |  | Aquifoliaceae | tree | wild | bark | mash and external apply | relieve pain，treat sore and furuncle | LC | 0.33 | 0.41 | YY210909031 |
|  |  |  |  |  | branches | decoction for medicinal bath |  |  |  |  |  |
| *Illicium dunnianum* Tutcher | shan ba jiao | Schisandraceae | shrub | wild | root | mash and external apply | treat rheumatism | LC | 0.02 | 0.00 | YY201209064 |
| *Illigera aromatica* S. Z. Huang & S. L. Mo |  | Hernandiaceae | liana | wild | branches | decoction for medicinal bath | remove dampness, treat dust allergy | LC | 0.13 | 0.00 | YY210911036 |
| *Illigera rhodantha* Hance | gou zhi teng | Hernandiaceae | liana | wild | stem | decoction for medicinal bath | treat rheumatism | LC | 0.10 | 0.14 | YY210118034 |
| *Impatiens clavigera* J. D. Hooker | chui feng san | Balsaminaceae | herb | wild | whole plant | decoction for medicinal bath | treat rheumatism | LC | 0.17 | 0.00 | YY201209070 |
|  |  |  |  |  | whole plant | decoction for oral administration | clear heat-toxin |  |  |  |  |
| *Isodon walkeri* (Arnott) H. Hara |  | Lamiaceae | herb | wild | whole plant | decoction for oral administration | treat hypertension, cool the blood, treat hepatitis, treat hepatitis B | LC | 0.19 | 0.23 | YY201208047 |
| *Ixonanthes reticulata* Jack |  | Ixonanthaceae | tree | wild | branches | decoction for medicinal bath | relieve fatigue, promote blood circulation and remove blood stasis, treat rheumatism | VU | 0.18 | 0.18 | YY210909007 |
| *Ixora finlaysoniana* Wallich ex G. Don | zhen dong mu | Rubiaceae | tree | wild | leaf | mash and external apply | treat tetanus | LC | 0.19 | 0.14 | YY210118018 |
| *Ixora henryi* H. Leveille | hei gou gu | Rubiaceae | shrub | wild | branches | mash and external apply, decoction for medicinal bath | relieve fatigue | LC | 0.21 | 0.45 | YY210118026 |
|  |  |  |  |  | root | mash and external apply | treat bone fracture |  |  |  |  |
|  |  |  |  |  | root, old stem | decoction for medicinal bath | treat rheumatism |  |  |  |  |
| *Jasminum lanceolaria* Roxburgh | tu yu dan | Oleaceae | shrub | wild | branches | decoction for medicinal bath | promote blood circulation and remove blood stasis, remove dampness, relieve itching, relieve fatigue, treat dust allergy | LC | 0.36 | 0.59 | YY210119013 |
|  |  |  |  |  | root | decoction for oral administration | gall-stone |  |  |  |  |
| *Juncus effusus* Linnaeus |  | Juncaceae | herb | wild | whole plant | decoction for oral administration, decoction for medicinal bath | calm the nerve, calm children's nerve | LC | 0.35 | 0.41 | YY210121014 |
| *Justicia adhatoda* Linnaeus |  | Acanthaceae | shrub | cultivated | branches | decoction for medicinal bath, medicinal wine for oral administration | treat rheumatism, treat bruises, treat sprain | LC | 0.18 | 0.09 | YY210122019 |
| *Justicia gendarussa* N. L. Burman |  | Acanthaceae | herb | wild | whole plant | mash and external apply, decoction for medicinal bath | treat rheumatism, treat sprain, treat bone fracture | LC | 0.22 | 0.23 | YY201209042 |
| *Kadsura coccinea* (Lemaire) A. C. Smith | fan tuan | Schisandraceae | liana | wild | leaf | decoction for medicinal bath | treat bruises | VU | 0.08 | 0.14 | YY201208015 |
|  |  |  |  |  | root | medicinal wine for oral administration | medicinal wine for oral administrationtreat gout, treat rheumatism |  |  |  |  |
| *Kyllinga brevifolia* Rottboll |  | Cyperaceae | herb | wild | whole plant | decoction for medicinal bath | treat bruises | LC | 0.23 | 0.23 | YY210121034 |
|  |  |  |  |  |  | mash and external apply | treat bone fracture |  |  |  |  |
| *Lantana camara* Linnaeus |  | Verbenaceae | shrub | wild | branches | decoction for medicinal bath | remove dampness, relieve itching, treat dust allergy | LC | 0.21 | 0.27 | HRC210907042 |
| *Lasia spinosa* (Linnaeus) Thwaites |  | Araceae | herb | wild | stem | decoction for oral administration | clear the heat | LC | 0.14 | 0.00 | YY210120004 |
| *Lasianthus verticillatus* (Loureiro) Merrill | hei gou gu | Rubiaceae | shrub | wild | rhizome | mash and external apply | treat bone fracture | LC | 0.03 | 0.00 | YY210908028 |
| *Lasiobema championii* (Benth.) de Wit | xiao zhong yang ti jia | Fabaceae | liana | wild | stem | decoction for medicinal bath | treat rheumatism | LC | 0.21 | 0.41 | YY210118032 |
| *Lemmaphyllum microphyllum* C.Presl |  | Polypodiaceae | herb | wild | whole plant | mash and external apply | treat herpes zoster | LC | 0.22 | 0.36 | YY210121041 |
| *Leonurus japonicus* Houttuyn |  | Lamiaceae | herb | wild | branches | decoction for oral administration | treat gynecological diseases | LC | 0.38 | 0.64 | YY210122016 |
| *Ligustrum sinense* Loureiro |  | Oleaceae | shrub | wild | branches | mash and external apply | treat headache | LC | 0.02 | 0.00 | YY201209091 |
| *Liparis stricklandiana* H. G. Reichenbach |  | Orchidaceae | herb | wild | whole plant | mash and external apply, decoction for oral administration | treat rheumatism | LC | 0.05 | 0.00 | YY210121052 |
|  |  |  |  |  |  | decoction for oral administration | clear the heat in lungs, treat hepatitis | LC | 0.05 | 0.00 | YY210121052 |
| *Liquidambar chingii* (F.P.Metcalf) Ickert-Bond & J.Wen | feng he gui | Altingiaceae | tree | wild | whole plant | medicinal wine for oral administration, medicinal wine for external application | treat rheumatism | VU | 0.14 | 0.27 | YY210910007 |
|  |  |  |  |  |  | decoction for medicinal bath | help postpartum recovery |  |  |  |  |
| *Liquidambar formosana* Hance |  | Altingiaceae | tree | wild | branches | decoction for medicinal bath | treat rheumatism, promote blood circulation and remove blood stasis, relieve fatigue, relieve muscle pain and headache, help postpartum recovery | LC | 0.60 | 0.91 | YY210909004 |
| *Litsea cubeba* (Loureiro) Persoon |  | Lauraceae | tree | wild | branches | decoction for medicinal bath | promote blood circulation and remove blood stasis, treat rheumatism, treat coldtreat fever, relieve fatigue, help postpartum recovery, relieve muscle pain and headaches | LC | 0.38 | 0.68 | HRC210907004 |
| *Lobelia zeylanica* Linnaeus |  | Campanulaceae | herb | wild | whole plant | mash and external apply | treat sore and furuncle | LC | 0.04 | 0.00 | YY210121045 |
| *Lonicera confusa* (Sweet) DC. |  | Caprifoliaceae | liana | wild | branches | decoction for medicinal bath | relieve fatigue, promote blood circulation and remove blood stasis, remove dampness, treat dust allergy | LC | 0.31 | 0.45 | YY210909002 |
| *Lonicera hypoglauca* Miquel | gu xian ren dong | Caprifoliaceae | liana | wild | branches | decoction for oral administration | clear heat-toxin | LC | 0.31 | 0.50 | YY210119056 |
|  |  |  |  |  |  | decoction for medicinal bath | miliaria |  |  |  |  |
| *Lonicera japonica* Thunberg in Murray |  | Caprifoliaceae | liana | wild | branches | decoction for medicinal bath | remove dampness and treat dust allergy | LC | 0.31 | 0.36 | YY210909019 |
| *Lonicera macrantha* Spreng. |  | Caprifoliaceae | liana | wild | flower | decoction for oral administration | clear heat-toxin | VU | 0.33 | 0.41 | YY210118043 |
| *Lophatherum gracile* Brongniart in Duperrey |  | Poaceae | herb | wild | whole plant | decoction for medicinal bath | promote blood circulation and remove blood stasis, relieve itching, relieve fatigue,calm children‘s nerve, treat coldtreat fever, treat cough | LC | 0.51 | 1.00 | HRC210907024 |
| *Ludwigia adscendens* (Linnaeus) H. Hara |  | Onagraceae | herb | wild | root | decoction for medicinal bath | prevent infantile colds, remove dampness | LC | 0.26 | 0.50 | HRC210907060 |
| *Luffa aegyptiaca* Miller |  | Cucurbitaceae | liana | cultivated | melon pulp | decoction for medicinal bath | relieve itching | LC | 0.16 | 0.18 | YY210121007 |
| *Luisia morsei* Rolfe | mu cong | Orchidaceae | herb | wild | whole plant | therapeutic diet | treat cervical spondylosis | LC | 0.03 | 0.00 | YY201209033 |
| *Lycopodium* L. |  | Lycopodiaceae | herb | wild | whole plant | decoction for medicinal bath | treat infantile colds and runny nose | LC | 0.25 | 0.23 | YY210911013 |
| *Lygodium flexuosum* (L.) Sw. |  | Lygodiaceae | herb | wild | whole plant | decoction for oral administration | gall-stone | LC | 0.65 | 0.95 | YY201209013 |
|  |  |  |  |  |  | decoction for medicinal bath | treat rheumatism, relieve itching, treat coldtreat fever, calm children's nerve |  |  |  |  |
| *Lygodium japonicum* (Thunb.) Sw. |  | Lygodiaceae | herb | wild | whole plant | decoction for medicinal bath | promote blood circulation and remove blood stasis, relieve itching, relieve fatigue | LC | 0.40 | 0.82 | HRC210907017 |
| *Macaranga indica* Wight | fan bao shu | Euphorbiaceae | tree | wild | root, myron | root:decoction for medicinal bath; myron:external apply | treat rheumatism, clean white tongue coating | LC | 0.10 | 0.14 | YY210118019 |
| *Maclura tricuspidata* Carriere |  | Moraceae | shrub | wild | root, stem | decoction for medicinal bath, decoction for oral administration | treat rheumatism, treat stone | LC | 0.33 | 0.41 | YY210118023 |
| *Macrosolen cochinchinensis* (Loureiro) Tieghem |  | Loranthaceae | shrub | wild | whole plant | decoction for oral administration | treat frequent urination | LC | 0.19 | 0.36 | YY210908009 |
| *Maesa acuminatissima* Merrill |  | Primulaceae | shrub | wild | branches | decoction for medicinal bath | clear heat and dispel dampness | LC | 0.33 | 0.59 | YY210120036 |
| *Maesa japonica* (Thunberg) Moritzi & Zollinger | qing qi | Primulaceae | shrub | wild | branches | decoction for medicinal bath | treat knife wounds and inflamed wounds | LC | 0.03 | 0.00 | YY210119008 |
| *Maesa montana* A. de Candolle |  | Primulaceae | shrub | wild | leaf | decoction for medicinal bath | treat wound inflammation, treat rheumatism | LC | 0.16 | 0.18 | YY210908006 |
|  |  |  |  |  | root | decoction for oral administration | treat sore throat |  |  |  |  |
| *Mallotus apelta* (Loureiro) Muller Argoviensis |  | Euphorbiaceae | tree | wild | branches | decoction for medicinal bath | treat rheumatism | LC | 0.18 | 0.55 | YY201209014 |
| *Mallotus paniculatus* (Lamarck) Muller Argoviensis | bai bei tong | Euphorbiaceae | tree | wild | branches | decoction for oral administration | dispel dampness and stop itching | LC | 0.30 | 0.73 | YY201208011 |
|  |  |  |  |  | tuber | decoction for medicinal bath | treat hepatitis, prevent infantile colds |  |  |  |  |
| *Manihot esculenta* Crantz |  | Euphorbiaceae | shrub | cultivated | tuberous root | mash and external apply | treat tetanus | LC | 0.02 | 0.00 | YY201208051 |
| *Melastoma dodecandrum* Loureiro |  | Melastomataceae | shrub | wild | whole plant | decoction for medicinal bath | remove dampness, relieve itching，treat dust allergy, treat cold, treat fever, runny nose | LC | 0.33 | 0.32 | YY201209027 |
|  |  |  |  |  |  | decoction with land plaster and then orally taken the liquid part | treat toothache |  |  |  |  |
| *Melastoma intermedium* Dunn | man di gong | Melastomataceae | shrub | wild | leaf, root | mash and external apply | reduce skin ulcer | LC | 0.09 | 0.00 | YY201209026 |
| *Melastoma malabathricum* Linnaeus |  | Melastomataceae | shrub | wild | branches | decoction for medicinal bath | treat infatile common cold | LC | 0.17 | 0.14 | YY201209018 |
|  |  |  |  |  |  | mash and external apply | stop bleeding | LC | 0.17 | 0.14 | YY201209018 |
| *Melastoma sanguineum* Sims |  | Melastomataceae | shrub | wild | branches | decoction for medicinal bath | treat infatile common cold, treat fever | LC | 0.32 | 0.27 | YY201209017 |
|  |  |  |  |  |  | mash and external apply | stop bleeding, treat sore and furuncle |  |  |  |  |
| *Melicope pteleifolia* (Champion ex Bentham) T. G. Hartley |  | Rutaceae | tree | wild | branches | mash and external apply | treat insect-bite | LC | 0.73 | 1.00 | YY201209003 |
|  |  |  |  |  |  | decoction for medicinal bath | reduce swellingrelieve pain, promote blood circulation and remove blood stasis, relieve itching, relieve fatigue, prevent the flu, treat cold, fever, skin ulcer |  |  |  |  |
|  |  |  |  |  |  | decoction for oral administration | treat cold, runny nose |  |  |  |  |
| *Melocalamus arrectus* T. P. Yi |  | Poaceae | herb | wild | branches | decoction for medicinal bath | relieve fatigue, promote blood circulation and remove blood stasis, exorcise evil spirits, dispel the wind, treat infantile common cold, runny nose | LC | 0.30 | 0.36 | YY210909009 |
| *Melodinus cochinchinensis* (Lour.) Merr. | hei mo tang | Apocynaceae | liana | wild | root | mash and external apply | treat pimple | LC | 0.06 | 0.00 | YY210908030 |
| *Microcos paniculata* Linnaeus | gua bu shu | Malvaceae | tree | wild | bark, branches | decoction for oral administration | treat pneumonia, treat hepatitis | LC | 0.42 | 0.68 | YY201209025 |
|  |  |  |  |  |  | decoction for medicinal bath | promote blood circulation and remove blood stasis, relieve fatigue, relieve itching |  |  |  |  |
| *Microglossa pyrifolia* (Lamarck) Kuntze |  | Asteraceae | herb | wild | branches | decoction for medicinal bath | treat gynecological diseases | LC | 0.03 | 0.00 | YY210531025 |
| *Micromelum integerrimum* (Buchanan-Hamilton ex Candolle) Wight & Arnott ex M. Roemer |  | Rutaceae | tree | wild | stem, leaf, root | stem: decoction for medicinal bath;leaf:mash and external apply; root:medicinal wine for external apply | treat rheumatism | LC | 0.20 | 0.36 | YY210118033 |
| *Micromelum minutum* (G.Forst.) Wight & Arn. | bai mu | Rutaceae | tree | wild | branches | decoction for medicinal bath | treat rheumatism | LC | 0.16 | 0.23 | YY201209097 |
| *Morinda officinalis* F. C. How |  | Rubiaceae | liana | wild | tuberous root | medicinal soup, medicinal wine for oral administration | treat rheumatism, nourish the body, tonify the kidney | VU | 0.27 | 0.59 | YY201209088 |
| *Mucuna birdwoodiana* Tutcher |  | Fabaceae | liana | wild | stem | medicinal soup | black hair | LC | 0.05 | 0.14 | YY201209101 |
| *Musa balbisiana* Colla | ye ba jiao | Musaceae | herb | wild | stem | decoction for oral administration | treat gynecological diseases | LC | 0.06 | 0.00 | YY210119051 |
| *Mussaenda erosa* Champion ex Bentham | da liang teng | Rubiaceae | shrub | wild | branches | mash and external apply | stop bleeding | LC | 0.31 | 0.00 | YY201209005 |
|  |  |  |  |  | whole plant | decoction for oral administration | clear heat-toxin |  |  |  |  |
| *Mussaenda pubescens* W. T. Aiton | xiao liang teng | Rubiaceae | liana | wild | branches | decoction for medicinal bath | cold, fever | LC | 0.47 | 0.64 | YY201208005 |
|  |  |  |  |  | whole plant | decoction for orally administration | clear heat-toxin , treat rheumatism |  |  |  |  |
|  |  |  |  |  |  | mash and external apply | treat knife wound, inflammation, treat sore and furuncle |  |  |  |  |
| *Nageia nagi* (Thunberg) Kuntze |  | Podocarpaceae | tree | wild | branches | mash and external apply | prevention of wound infection and inflammation | EN | 0.01 | 0.00 | YY201209069 |
| *Naravelia pilulifera* Hance | lao hu xu | Ranunculaceae | liana | wild | whole plant | decoction for medicinal bath | treat rheumatism | LC | 0.05 | 0.00 | YY210908018 |
| *Nephelaphyllum tenuiflorum* Blume | guo shan feng | Orchidaceae | herb | wild | whole plant | decoction for medicinal bath, decoction for oral administration | treat rheumatism | VU | 0.03 | 0.00 | YY201209048 |
| *Nephrolepis cordifolia* (L.) C. Presl | shi dan zi | Nephrolepidaceae | herb | wild | root | orally taken directly | treat sphagitis | LC | 0.38 | 0.41 | YY210120017 |
|  |  |  |  |  | tuber | orally taken directly | treat hepatitis, treat nephritis，diuresis |  |  |  |  |
| *Odontochilus elwesii* C. B. Clarke ex J. D. Hooker |  | Orchidaceae | herb | wild | whole plant | medicinal soup | nourish the body | LC | 0.01 | 0.00 | YY201209085 |
| *Odontosoria chinensis* (L.) J.Sm. | feng huang cao | Lindsaeaceae | herb | wild | whole plant | decoction for oral administration | clear heat-toxin | LC | 0.13 | 0.27 | YY201209095 |
| *Ohwia caudata* (Thunberg) H. Ohashi | dong ma huang, san ye qing | Fabaceae | shrub | wild | leaf | mash and taken with rice wine | treat snakebites | LC | 0.19 | 0.14 | YY201208061 |
| *Oldenlandia hedyotidea* (DC.) Hand.-Mazz. |  | Rubiaceae | shrub | wild | whole plant | decoction for medicinal bath | clear heat-toxin | LC | 0.36 | 0.41 | YY201209007 |
| *Oldenlandia platystipula* (Merr.) Chun |  | Rubiaceae | herb | wild | branches | decoction for medicinal bath | treat rheumatism, treat neonatal skin infection | LC | 0.27 | 0.36 | YY210601001 |
| *Ophiopogon chingii* F. T. Wang & Tang | shan jiu cai | Asparagaceae | herb | wild | root | mash and external apply | promote blood circulation and remove blood stasis，treat sprain | LC | 0.04 | 0.00 | YY210119043 |
|  |  |  |  |  | whole plant | decoction for oral administration | treat hepatitis B |  |  |  |  |
| *Ophiopogon sylvicola* F. T. Wang & Tang | shan jiu cai | Asparagaceae | herb | wild | whole plant | decoction for medicinal bath | reduce swelling and relieve pain | NT | 0.01 | 0.00 | YY210118017 |
| *Oryza sativa* L. |  | Poaceae | herb | cultivated | seed | mash and external apply | treat herpes zoster | LC | 0.01 | 0.00 | YY210118048 |
| *Osbeckia opipara* C. Y. Wu et C. Chen |  | Melastomataceae | shrub | wild | whole plant | decoction for medicinal bath | promote blood circulation and remove blood stasis, treat rheumatism | LC | 0.06 | 0.00 | YY210119002 |
| *Oxalis corniculata* Linnaeus |  | Oxalidaceae | herb | wild | whole plant | mash and external apply | treat sore and furuncle | LC | 0.17 | 0.00 | YY210121035 |
| *Oxalis debilis* Kunth |  | Oxalidaceae | herb | wild | whole plant | mash and external apply | treat sore and furuncle | LC | 0.19 | 0.23 | YY210121036 |
| *Paederia foetida* L. |  | Rubiaceae | liana | wild | leaf | decoction for medicinal bath | treat gynecological diseases, clear heat-toxin | LC | 0.31 | 0.36 | YY210122010 |
| *Lycopodiella cernua* (L.) Pic.Serm. | shen jin cao | Lycopodiaceae | herb | wild | whole plant | decoction for medicinal bath | promote blood circulation and remove blood stasis, treat rheumatism, exorcise evil spirits, calm children's nerve, treat infantile common cold, relieve itching, relieve fatigue | LC | 0.50 | 1.00 | YY201209094 |
| *Pandanus austrosinensis* T. L. Wu | lei gu | Pandanaceae | herb | wild | root, fruit | decoction for oral administration | treat hepatitis, treat headache, inflammation, treat hypertension | LC | 0.19 | 0.32 | YY210120020 |
| *Paris fargesii* Franchet |  | Melanthiaceae | herb | wild | tuber | mash and external apply | treat snake bites | NT | 0.06 | 0.00 | YY201209052 |
| *Paris polyphylla* Smith in Rees | hong zong ye | Melanthiaceae | herb | cultivated | tuber | mash and external apply | treat sore and furuncle, treat snake bites, treat cervical or lumbar sprains | NT | 0.22 | 0.14 | YY201208063 |
| *Parthenocissus* dalzielii Gagnepain |  | Vitaceae | liana | wild | whole plant | decoction for medicinal bath | treat migratory viral infection | LC | 0.02 | 0.00 | YY201209074 |
| *Parthenocissus tricuspidata* (Siebold & Zuccarini) Planchon in A. Candolle & C. Candolle |  | Vitaceae | liana | wild | whole plant | mash and external apply | treat herpes zoster | LC | 0.08 | 0.00 | HRC210906004 |
| *Pentaphragma spicatum* Merr. |  | Pentaphragmataceae | herb | wild | whole plant | decoction for medicinal bath | treat rheumatism, treat bruises, promote blood circulation and remove blood stasis | DD | 0.26 | 0.14 | YY210121048 |
| *Perilla frutescens* (Linnaeus) Britton |  | Lamiaceae | herb | cultivated | branches | decoction for medicinal bath | promote blood circulation and remove blood stasis, loosen tendons and activate collaterals, relieve fatigue，treat infantile common coldrunny nose | LC | 0.34 | 0.55 | YY210121031 |
|  |  |  |  |  | whole plant | decoction for oral administration | heat in lungs |  |  |  |  |
| *Persicaria chinensis* (L.) H. Gross |  | Polygonaceae | herb | wild | whole plant | decoction for oral administration, decoction for medicinal bath | clear heat-toxin , relieve itching, treat measles | LC | 0.50 | 0.73 | YY201209016 |
| *Phanera erythropoda* (Hayata) Mackinder & R.Clark |  | Fabaceae | liana | wild | branches | decoction for medicinal bath | relieve itching | LC | 0.05 | 0.00 | YY201209102 |
| *Phanera ornata* var. *balansae* (Gagnep.) Bandyop., Ghoshal & M.K.Pathak |  | Fabaceae | tree | wild | branches | decoction for medicinal bath | remove dampness, relieve itching | LC | 0.16 | 0.27 | HRC210907041 |
| *Phanera ornata* var. *kerrii* (Gagnep.) Bandyop., Ghoshal & M.K.Pathak |  | Fabaceae | herb | wild | branches | decoction for medicinal bath | treat skin diseases, relieve itching | LC | 0.06 | 0.00 | YY210118014 |
|  |  |  |  |  | stem | decoction for oral administration | treat rheumatism, promote blood circulation and remove blood stasis |  |  |  |  |
| *Pholidota chinensis* Lindley |  | Orchidaceae | herb | wild | whole plant | decoction for oral administration | clear away heat and remove phlegm, treat cough, and tuberculosis | LC | 0.44 | 0.77 | YY201209045 |
| *Phragmites australis* (Cav.) Steud. |  | Poaceae | herb | wild | tuber | decoction for oral administration, decoction for medicinal bath | treat hepatitis, relieve pain and detumescence | LC | 0.26 | 0.00 | YY201209082 |
| *Phyllanthus emblica* Linnaeus |  | Phyllanthaceae | tree | wild | leaf | decoction for medicinal bath | calm children' nerve, treat fever | LC | 0.06 | 0.00 | YY210531011 |
| *Phyllanthus eriocarpus* (Champ. ex Benth.) Müll.Arg. |  | Phyllanthaceae | shrub | wild | branches | decoction for medicinal bath | remove dampness, stop itching, treat dust allergy | LC | 0.36 | 0.73 | YY201209009 |
| *Phyllanthus urinaria* Linnaeus |  | Phyllanthaceae | herb | wild | whole plant | decoction for oral administration | treat infantile malnutrition | LC | 0.19 | 0.14 | YY210121023 |
|  |  |  |  |  |  | decoction for medicinal bath | treat infantile malnutrition |  |  |  |  |
| *Phyllanthus niruri* L. | bai bei jing | Phyllanthaceae | herb | wild | whole plant | decoction for medicinal bath | treat infantile malnutrition | LC | 0.13 | 0.09 | HRC210906005 |
| *Phymatosorus cuspidatus* (D. Don) Pic. Serm. | she qing | Polypodiaceae | herb | wild | stem | mash and external apply | treat snake bites | LC | 0.16 | 0.23 | YY210908054 |
|  |  |  |  |  |  | decoction for medicinal bath | treat rheumatism |  |  |  |  |
| *Physalis angulata* L. |  | Solanaceae | herb | wild | whole plant | decoction for medicinal bath | dispel dampness and stop itching | LC | 0.19 | 0.27 | HRC210907038 |
| *Pinus massoniana* Lambert |  | Pinaceae | tree | wild | branches | decoction for medicinal bath | remove dampness, relieve itching | LC | 0.26 | 0.36 | HRC210907032 |
| *Piper sarmentosum* Roxb. | jia lou | Piperaceae | herb | wild | leaf | mash and external apply | promote blood circulation and remove blood stasis, treat bruises | LC | 0.31 | 0.27 | YY201208068 |
| *Piper semiimmersum* C. de Candolle | zhong feng tou | Piperaceae | liana | wild | branches | decoction for medicinal bath | treat arthralgia, treat bruises, promote blood circulation and remove blood stasis | LC | 0.08 | 0.00 | YY201209065 |
| *Piper sintenense* Hatusima | chu shan hu | Piperaceae | liana | wild | whole plant | mash and external apply | treat sprain, treat scald | LC | 0.11 | 0.00 | YY201209041 |
| *Pittosporum glabratum* Lindley | shan la jiao | Pittosporaceae | shrub | wild | stem, root | decoction for medicinal bath | treat rheumatism | LC | 0.17 | 0.41 | YY210118048 |
| *Plantago asiatica* Linnaeus | dian xian cao | Plantaginaceae | herb | wild | whole plant | decoction for oral administration | clear inner heat, help diuresis | LC | 0.64 | 1.00 | YY210122014 |
| *Platostoma palustre* (Blume) A. J. Paton |  | Lamiaceae | herb | wild | whole plant | decoction for oral administration | treat hypertension | LC | 0.36 | 0.32 | YY210121044 |
| *Pleioblastus amarus* (Keng) P. C. Keng |  | Poaceae | herb | wild | branches | decoction for medicinal bath | treat cold and fever, dispel dampness | LC | 0.05 | 0.00 | HRC210907013 |
| *Pleocnemia submembranacea* (Hayata) Tagawa & K.Iwats. |  | Polypodiaceae | herb | wild | stem | medicinal wine for oral administration | treat rheumatism | LC | 0.01 | 0.00 | YY210118052 |
| Plumbago zeylanica Linnaeus |  | Plumbaginaceae | shrub | wild | branches | mash and external apply | treat hyperosteogeny | LC | 0.14 | 0.32 | YY201208045 |
| *Podophyllum versipelle* Hance |  | Berberidaceae | herb | wild | tuber | mash and external apply | treat haemorrhoids, treat osteoproliferation, treat rheumatism, treat bruises | VU | 0.15 | 0.14 | YY201209079 |
| *Pogonatherum crinitum* (Thunberg) Kunth |  | Poaceae | herb | wild | whole plant | decoction for oral administration | clear heat-toxin | LC | 0.24 | 0.23 | YY210601011 |
| *Pogonatherum paniceum* (Lamarck) Hackel |  | Poaceae | herb | wild | whole plant | decoction for oral administration | clear heat-toxin , treat hypertension | LC | 0.31 | 0.27 | YY210601012 |
| *Polygala chinensis* Linnaeus |  | Polygalaceae | herb | wild | whole plant | decoction for oral administration | treat infantile malnutrition | LC | 0.19 | 0.23 | YY201208027 |
| *Polygala japonica* Houttuyn |  | Polygalaceae | herb | wild | whole plant | decoction for oral administration | treat infantile malnutrition | LC | 0.13 | 0.14 | YY210121025 |
| *Polygonum perfoliatum* L. |  | Polygonaceae | herb | wild | whole plant | decoction for medicinal bath | remove dampness, treat dust allergy and relieve itching | LC | 0.40 | 0.86 | YY210909016 |
| *Pothos chinensis* (Rafinesque) Merrill | tie wu gong | Araceae | liana | wild | whole plant | decoction for medicinal bath | treat rheumatism, promote blood circulation and remove blood stasis | LC | 0.31 | 0.45 | YY210908053 |
| *Pouzolzia zeylanica* (Linnaeus) Bennett |  | Urticaceae | herb | wild | branches | decoction for medicinal bath | treat fever, treat measles | LC | 0.09 | 0.00 | YY210531001 |
| *Premna puberula* var. *bodinieri* (H. Leveille) C. Y. Wu & S. Y. Pao | zhong feng tou | Lamiaceae | shrub | wild | whole plant | decoction for oral administration, decoction for medicinal bath | reduce edema | LC | 0.10 | 0.14 | YY210119067 |
| *Prunus persica* L. |  | Rosaceae | tree | cultivated | stem | decoction for medicinal bath | calm children' nerve, treat fever | LC | 0.40 | 0.68 | YY210531012 |
| *Psidium guajava* Linnaeus |  | Myrtaceae | tree | wild | tender shoot | decoction for oral administration | treat diarrhea | LC | 0.29 | 0.36 | YY210910004 |
| *Psychotria asiatica* Linnaeus | dao gu gang | Rubiaceae | shrub | wild | root | mash and external apply | treat bruises, stop bleeding, help wound healing, detoxificationcoldtreat fever等 | LC | 0.44 | 0.82 | YY201209001 |
|  |  |  |  |  |  | decoction for medicinal bath | remove dampness, relieve itching, promote blood circulation and remove blood stasis, relieve fatigue，treat coldtreat fever |  |  |  |  |
| *Psychotria serpens* Linnaeus | pai si bie | Rubiaceae | liana | wild | whole plant | decoction for medicinal bath | promote blood circulation and remove blood stasis, treat rheumatism, treat herpes zoster | LC | 0.27 | 0.27 | YY210908008 |
| *Pteris ensiformis* Burm. |  | Pteridaceae | herb | wild | whole plant | decoction for oral administration | treat diarrhea | LC | 0.08 | 0.00 | YY210120011 |
| *Pteris fauriei* Hieron. var. *fauriei* |  | Pteridaceae | herb | wild | leaf | decoction for oral administration | treat diarrhea, treat dysentery | LC | 0.01 | 0.00 | YY201208022 |
| *Pteris semipinnata* L. |  | Pteridaceae | herb | wild | leaf | mash and external apply | stop bleeding | LC | 0.11 | 0.23 | YY210120012 |
|  |  |  |  |  |  | decoction for oral administration | treat dysentery |  |  |  |  |
| *Pterospermum truncatolobatum* Gagnepain |  | Sterculiaceae | tree | wild | branches | decoction for oral administration, decoction for medicinal bath | treat rheumatism, treat measles | LC | 0.08 | 0.00 | YY210121026 |
| *Pueraria montana* (Loureiro) Merrill |  | Fabaceae | liana | wild | tuberous root | decoction for oral administration | treat rheumatism | LC | 0.30 | 0.18 | YY210118020 |
| *Pyrrosia lingua* (Thunb.) Farwell | sha ging | Polypodiaceae | herb | wild | whole plant | decoction for oral administration | treat diabetes, treat pulmonary edema, help diuresis | LC | 0.23 | 0.23 | YY210908036 |
| *Radermachera hainanensis* Merrill |  | Bignoniaceae | tree | wild | bark | decoction for medicinal bath | treat infantile body deficiency | LC | 0.14 | 0.14 | YY210118006 |
| *Rauvolfia serpentina* (Linnaeus) Bentham ex Kurz | huo you cai , chou qian li | Apocynaceae | shrub | wild | leaf | mash and external apply | detoxify, treat bruises, treat lumbar hyperplasia | VU | 0.03 | 0.00 | YY201208048 |
| *Rauvolfia vomitoria* Afzelius | shan xiong dan | Apocynaceae | shrub | wild | root | decoction for oral administration | treat bruises, promote blood circulation and remove blood stasis | NT | 0.05 | 0.00 | YY210119077 |
| *Reynoutria japonica* Houttuyn |  | Polygonaceae | herb | wild | branches | decoction for medicinal bath | remove dampness, treat dust allergy | LC | 0.38 | 0.32 | YY210909023 |
| *Rhaphidophora decursiva* (Roxburgh) Schott | xue liang san | Araceae | liana | wild | branches, stem | therapeutic diet | treat shaking hands | LC | 0.06 | 0.00 | YY201209072 |
|  |  |  |  |  | stem | decoction for medicinal bath, decoction for oral administration | treat rheumatism |  |  |  |  |
| *Rhaphidophora hongkongensis* Schott | shan wu gong, wu gong teng | Araceae | liana | wild | stem | decoction for oral administration | treat pulmonary tuberculosis | LC | 0.27 | 0.27 | YY201208012 |
|  |  |  |  |  | whole plant | decoction for medicinal bath | treat rheumatism |  |  |  |  |
| *Rhodomyrtus tomentosa* (Aiton) Hasskarl | nian zi mu | Myrtaceae | shrub | wild | gum | mash and external apply | treat scald, remove scars | LC | 0.44 | 0.73 | YY210120033 |
|  |  |  |  |  | root | decoction for oral administration | treat anemia；tender shoot |  |  |  |  |
|  |  |  |  |  | tender shoot | eat directly | stop diarrhea, treat diarrhea |  |  |  |  |
| *Rhus chinensis* Mill. var. *roxburghii* (DC.) Rehder |  | Anacardiaceae | tree | wild | branches | decoction for medicinal bath | treat rheumatism, relieve fatigue, promote blood circulation and remove blood stasis, treat infantile common cold, runny nose | LC | 0.55 | 1.00 | YY210601008 |
| *Ricinus communis* Linnaeus | bi ma | Euphorbiaceae | herb | wild | leaf | mash and external apply | treat infantile common coldrunny nose | LC | 0.27 | 0.55 | YY210119061 |
|  |  |  |  |  | stem | decoction for oral administration | treat haemorrhoids；leafmash and external applytreat tetanus |  |  |  |  |
| *Rourea microphylla* (Hooker & Arnott) Planchon | hong ye teng , shui teng | Connaraceae | shrub | wild | stem, root | decoction for oral administration | treat haemorrhoids, treat pulmonary edema | LC | 0.09 | 0.00 | YY201208004 |
| *Rubus alceifolius* Poiret in Lamarck | tuo pan ci | Rosaceae | shrub | wild | root | decoction for oral administration | treat hepatitis B | LC | 0.04 | 0.00 | YY210119039 |
|  |  |  |  |  |  | decoction for medicinal bath | treat stomatitis |  |  |  |  |
| *Rubus parvifolius* Linnaeus | san dian jin | Rosaceae | shrub | wild | root | decoction for oral administration, decoction for medicinal bath | treat gynecological diseases | LC | 0.08 | 0.00 | YY201209022 |
| *Rubus rosifolius* Smith |  | Rosaceae | shrub | wild | root | decoction for medicinal bath | treat ophthalmology such as pinkeye | LC | 0.06 | 0.00 | YY210121043 |
| *Sarcandra glabra* (Thunberg) Nakai |  | Chloranthaceae | shrub | wild | whole plant | decoction for oral administration, medicinal wine for oral administration | treat joint pain, treat rheumatism, treat sore throat | LC | 0.57 | 0.91 | YY201209086 |
| *Saurauia tristyla* Candolle | bai fan guo | Actinidiaceae | tree | wild | root | decoction for oral administration, medicinal wine for oral administration | treat stomachache, treat senile dementia | LC | 0.05 | 0.00 | YY210908065 |
| *Schefflera arboricola* (Hayata) Merrill | qi ye feng | Araliaceae | shrub | wild | branches | decoction for medicinal bath | treat rheumatism, treat dust allergy | LC | 0.19 | 0.27 | YY201209035 |
| *Schefflera elliptica* (Blume) Harms in Engl. & Prantl | dong zhang feng | Araliaceae | shrub | wild | branches | decoction for medicinal bath | help postpartum recovery, remove dampness | LC | 0.30 | 0.55 | YY201209061 |
| *Schefflera heptaphylla* (Linnaeus) Frodin |  | Araliaceae | tree | wild | leaf, stem, root | decoction for medicinal bath, medicinal wine for external application | treat rheumatism, treat bruises, promote blood circulation and remove blood stasis, relieve fatigue, treat coldtreat fever | LC | 0.44 | 0.77 | HRC210907005 |
| *Schefflera pes-avis* R. Vig |  | Araliaceae | tree | wild | branches | decoction for oral administration, decoction for medicinal bath | treat rheumatism, treat measles | DD | 0.08 | 0.00 | YY210121029 |
| *Scolopia chinensis* (Loureiro) Clos | hong sheng nan | Salicaceae | tree | wild | tuber | decoction for oral administration | treat kidney and urinary calculi | LC | 0.04 | 0.00 | YY210908041 |
| *Scoparia dulcis* Linnaeus |  | Plantaginaceae | herb | wild | whole plant | decoction for oral administration | treat diabetes | LC | 0.33 | 0.41 | YY210122005 |
| *Securidaca inappendiculata* Hasskarl |  | Polygalaceae | shrub | wild | branches | decoction for medicinal bath | relieve fatigue, promote blood circulation and remove blood stasis | LC | 0.13 | 0.14 | YY210909008 |
|  |  |  |  |  | root | decoction for oral administration | nourish the body, tonify the kidney，treat hepatitis B, treat rheumatism |  |  |  |  |
| *Selaginella uncinata* (Desv.) Spring |  | Selaginellaceae | herb | wild | whole plant | decoction for oral administration | treat hepatitis | LC | 0.19 | 0.14 | YY210121046 |
| *Senecio scandens* Buchanan-Hamilton ex D. Don |  | Asteraceae | herb | wild | whole plant | decoction for medicinal bath | remove dampness, treat dust allergy | LC | 0.48 | 0.77 | YY210909022 |
| *Senecio vulgaris* Linnaeus | pu gong ying | Asteraceae | herb | wild | leaf | decoction for oral administration | reduce inflammation | LC | 0.06 | 0.00 | YY210122013 |
| *Smilax glabra* Roxb. |  | Smilacaceae | shrub | wild | tuber | decoction for oral administration | treat hepatitis B, tonify the kidney | LC | 0.29 | 0.27 | YY201208052 |
| *Smilax riparia* A. de Candolle in A. de Candolle & C. de Candolle |  | Smilacaceae | shrub | wild | old leaf | decoction for oral administration | loosen tendons and activate collaterals | LC | 0.03 | 0.00 | YY210122001 |
| *Solanum torvum* Swartz |  | Solanaceae | shrub | wild | leaf, root | mash and external apply | treat sore and furuncle | LC | 0.16 | 0.27 | YY210120005 |
| *Sonerila cantonensis* Stapf |  | Melastomataceae | herb | wild | branches | decoction for medicinal bath | treat rheumatism | LC | 0.08 | 0.23 | YY210601002 |
| *Spatholobus suberectus* Dunn |  | Fabaceae | liana | wild | branches | decoction for medicinal bath | treat rheumatism | VU | 0.29 | 0.36 | YY210122002 |
|  |  |  |  |  | stem | therapeutic diet | enrich the blood |  |  |  |  |
| *Sphaeromorphaea australis* (Less.) Kitam. |  | Asteraceae | herb | wild | whole plant | decoction for medicinal bath | reduce inflammation and treat measles | LC | 0.47 | 0.68 | HRC210906002 |
| *Staphylea japonica* (Thunb.) Mabb. |  | Staphyleaceae | tree | wild | branches | decoction for medicinal bath | reduce swelling, relieve itching | LC | 0.13 | 0.00 | YY201209040 |
|  |  |  |  |  | leaf | mash and external apply | help would healing |  |  |  |  |
| *Stemona tuberosa* Loureiro |  | Stemonaceae | herb | wild | tuberous root | mash with sulphur and external apply | treat tinea and sore | LC | 0.08 | 0.00 | YY201209060 |
|  |  |  |  |  |  | decoction for oral administration | treat pneumonia, treat pulmonary edema, treat cough |  |  |  |  |
| *Stephania cephalantha* Hayata |  | Menispermaceae | liana | wild | tuber | mash and external apply | treat sore and furuncle | LC | 0.16 | 0.27 | YY210121053 |
| *Stephania longa* Loureiro | jin xian feng | Menispermaceae | liana | wild | leaf | decoction for oral administration | treat rheumatism | LC | 0.26 | 0.41 | YY210119012 |
|  |  |  |  |  | tuberous root | chewing and taken orally, decoction for oral administration | treat sore throat |  |  |  |  |
| *Streptocaulon juventas* (Loureiro) Merrill |  | Apocynaceae | liana | wild | root | decoction for oral administration | promote lactation | LC | 0.03 | 0.00 | YY210119057 |
| *Strobilanthes cusia* (Nees) Kuntze |  | Acanthaceae | herb | cultivated | whole plant | decoction for oral administration | clear heat-toxin | LC | 0.07 | 0.00 | YY210910003 |
| *Stromanthe thalia* (Vell.) J.M.A.Braga | hong zhong ye | Marantaceae | herb | cultivated | stem | decoction for oral administration | Relieve alcohol and treat alcoholic liver | LC | 0.01 | 0.00 | YY210119079 |
| *Strophanthus divaricatus* (Loureiro) Hooker & Arnott | yang jiao niu | Apocynaceae | shrub | wild | leaf | decoction for medicinal bath | treat bruises, hand-foot-and-mouth disease, treat syphilis, eczema | LC | 0.18 | 0.27 | YY210119018 |
|  |  |  |  |  | whole plant | mash and external apply | treat sore and furuncle |  |  |  |  |
| *Symplocos paniculata* (Thunberg) Miquel |  | Symplocaceae | shrub | wild | root | mash and external apply | treat sore and furuncle | LC | 0.06 | 0.00 | YY201209023 |
| *Syzygium championii* (Bentham) Merrill & L. M. Perry | jiu pa mu | Myrtaceae | tree | wild | leaf | decoction for medicinal bath | treat rheumatism | LC | 0.20 | 0.18 | YY210908044 |
| *Syzygium hancei* Merrill & L. M. Perry | huo tan mu | Myrtaceae | tree | wild | branches | decoction for medicinal bath | relieve itching，treat tinea capitis | LC | 0.08 | 0.18 | YY201209037 |
|  |  |  |  |  |  | decoction for oral administration | treat diarrhea |  |  |  |  |
| *Syzygium jambos* (Linnaeus) Alston | shui fan tao | Myrtaceae | tree | wild | branches | decoction for medicinal bath | treat rheumatism | DD | 0.33 | 0.23 | YY210120028 |
| *Syzygium nervosum* Candolle |  | Myrtaceae | tree | wild | branches | decoction for oral administration | treat beriberi, tinea pedis | LC | 0.41 | 0.82 | YY210911009 |
|  |  |  |  |  | inflorescence, root | decoction for oral administration | clear heat-toxin ，treat infantile common coldrunny nose |  |  |  |  |
| *Tacca chantrieri* Andre | shui luo bo | Dioscoreaceae | herb | wild | whole plant | decoction for oral administration | clear heat-toxin | NT | 0.14 | 0.14 | YY210119080 |
| *Tacca plantaginea* (Hance) Drenth | shui tian qi | Dioscoreaceae | herb | wild | leaf | therapeautic diet | treat amenorrhea | LC | 0.16 | 0.18 | YY201208057 |
|  |  |  |  |  | whole plant | decoction for oral administration | clear heat-toxin |  |  |  |  |
| *Tadehagi triquetrum* (Linnaeus) H. Ohashi |  | Fabaceae | shrub | wild | branches | decoction for medicinal bath | remove dampness, relieve itching | LC | 0.41 | 0.59 | YY210908002 |
|  |  |  |  |  | leaf | mash and external apply | insect prevention |  |  |  |  |
|  |  |  |  |  | root | decoction for oral administration | clear inner heat, help diuresis，treat hepatitis, treat hepatitis B, treat inner heat, liver pain, high cholesterolitching |  |  |  |  |
| *Tainia dunnii* Rolfe |  | Orchidaceae | herb | wild | bulb | mash and external apply | treat sore and furuncle | NT | 0.02 | 0.00 | YY210121058 |
|  |  |  |  |  |  | decoction for oral administration | treat cough |  |  |  |  |
| *Tarenna depauperata* Hutchinson in Sargent | mu ben yi dian hong | Rubiaceae | tree | wild | root | decoction for medicinal bath | treat rheumatism, treat bruises | LC | 0.07 | 0.00 | YY210118037 |
| *Taxillus chinensis* (Candolle) Danser | jiu jie sang ji sheng | Loranthaceae | shrub | wild | whole plant | decoction for medicinal bath, medicinal wine for external application, medicinal wine for oral administration | treat bruises, promote blood circulation and remove blood stasis | LC | 0.27 | 0.41 | YY201209002 |
| *Tetracera sarmentosa* (Linnaeus) Vahl | hei gu teng | Dilleniaceae | liana | wild | root | decoction for oral administration | treat diarrhea, clear the throat | LC | 0.16 | 0.14 | YY201209099 |
| *Tetrastigma planicaule* (J. D. Hooker) Gagnepain |  | Vitaceae | liana | wild | stem, root | decoction for medicinal bath | treat rheumatism | LC | 0.13 | 0.00 | YY201209100 |
|  |  |  |  |  |  | medicinal wine for oral administration | treat nephritis, treat hepatitis |  |  |  |  |
| *Tinomiscium petiolare* Miers ex J. D. Hooker & Thomson |  | Menispermaceae | liana | wild | root | decoction for oral administration | treat rheumatism，treat sore throat, treat toothache | LC | 0.16 | 0.27 | YY201209068 |
|  |  |  |  |  | whole plant | decoction for medicinal bath | treat cold |  |  |  |  |
| *Tinospora sagittata* (Oliver) Gagnepain |  | Menispermaceae | liana | wild | tuberous root | direct administration | treat vomiting, treat diarrhea | EN | 0.29 | 0.23 | YY201209083 |
| *Tinospora sinensis* (Loureiro) Merrill | song jin teng | Menispermaceae | liana | wild | vine | decoction for oral administration, medicinal wine for external application, medicinal wine for oral administration | treat bruises, treat rheumatism | LC | 0.16 | 0.41 | YY210120034 |
| *Toddalia asiatica* (Linnaeus) Lamarck | liang mian zhen | Rutaceae | liana | wild | root | decoction for washing mouth | treat toothache | LC | 0.16 | 0.27 | YY210119016 |
| *Toxicodendron succedaneum* (Linnaeus) Kuntze |  | Anacardiaceae | tree | wild | tender shoot | mash and external apply | stop bleeding | LC | 0.02 | 0.00 | YY210910016 |
| *Toxicodendron vernicifluum* (Stokes) F. A. Barkley |  | Anacardiaceae | tree | wild | stem pith | therapeutic diet | relieve stomache | LC | 0.02 | 0.00 | YY210120019 |
| *Tradescantia zebrina* Bosse |  | Commelinaceae | herb | wild | whole plant | mash and external apply | treat bruises, wounds | LC | 0.14 | 0.00 | YY210531003 |
| *Trema cannabina* var. *dielsiana* (Handel-Mazzetti) C. J. Chen |  | Cannabaceae | herb | wild | branches | decoction for medicinal bath | treat measles | LC | 0.09 | 0.14 | HRC210907082 |
| *Trema tomentosa* (Roxburgh) H. Hara |  | Cannabaceae | tree | wild | stem, leaf | decoction for medicinal bath | treat measles | LC | 0.13 | 0.00 | YY201208042 |
| *Triadica cochinchinensis* Loureiro |  | Euphorbiaceae | shrub | wild | branches, stem | decoction for medicinal bath | remove dampness，treat dust allergy, gynecological diseases | LC | 0.19 | 0.23 | YY210909025 |
|  |  |  |  |  | root | decoction for oral administration | treat constipation |  |  |  |  |
| *Triadica sebifera* (Linnaeus) Small |  | Euphorbiaceae | tree | wild | stem | decoction for medicinal bath | treat gynecological diseases | LC | 0.17 | 0.32 | YY210531022 |
| *Trichosanthes truncata* C. B. Clarke |  | Cucurbitaceae | liana | wild | leaf | mash and external apply | treat sore and furuncle | LC | 0.13 | 0.00 | YY210121054 |
| *Triumfetta cana* Blume |  | Malvaceae | herb | wild | leaf | mash and cover nose | treat allergic rhinitis | LC | 0.02 | 0.00 | YY210119047 |
| Tropidia nipponica Masamune |  | Orchidaceae | herb | wild | root | mash and external apply | promote blood circulation and remove blood stasis | NT | 0.01 | 0.00 | YY210908061 |
| *Uncaria hirsuta* Haviland |  | Rubiaceae | liana | wild | whole plant | decoction for medicinal bath | treat rheumatism, treat measles, help postpartum recovery | LC | 0.29 | 0.36 | YY201209104 |
| *Uncaria sinensis* (Oliver) Haviland |  | Rubiaceae | liana | wild | branches | decoction for medicinal bath | relieve fatigue, relieve muscle pain, promote blood circulation and remove blood stasis,treat rheumatism, treat headache, help postpartum recovery | LC | 0.28 | 0.27 | HRC210907046 |
| *Urceola huaitingii* (Chun & Tsiang) D. J. Middleton | dong zhong teng | Apocynaceae | shrub | wild | bark | medicinal wine for oral administration | treat rheumatism, tonify bones | LC | 0.06 | 0.00 | YY210120014 |
| *Urceola micrantha* (Wallich ex G. Don) D. J. Middleton |  | Apocynaceae | shrub | wild | root bark | decoction for oral administration | tonify the kidney | LC | 0.13 | 0.00 | YY210118046 |
|  |  |  |  |  | stem | decoction for medicinal bath | treat rheumatism |  |  |  |  |
| *Urena procumbens* Linnaeus |  | Malvaceae | shrub | wild | leaf | mash and external apply | treat bone fracture | LC | 0.13 | 0.00 | YY210120010 |
| *Uvaria littoralis* Blume | teng ba jiu | Annonaceae | shrub | wild | root | mash and external apply | promote blood circulation and remove blood stasis | LC | 0.17 | 0.23 | YY210908052 |
| *Ventilago leiocarpa* Bentham | qing teng | Rhamnaceae | shrub | wild | whole plant, leaf, root | decoction for oral administration, decoction for medicinal bath | clear heat-toxin , treat colds and hepatitis | LC | 0.19 | 0.32 | YY210118009 |
| *Verbena officinalis* Linnaeus |  | Verbenaceae | herb | wild | whole plant | decoction for oral administration | treat infantile fever | LC | 0.27 | 0.23 | YY210531013 |
|  |  |  |  |  |  | decoction for medicinal bath | treat gynecological diseases |  |  |  |  |
| *Viburnum fordiae* Hance | niu shou bian | Adoxaceae | shrub | wild | leaf | mash and external apply | treat sore and furuncle, relieve pain, treat sore and furuncle | LC | 0.07 | 0.00 | YY201208007 |
| *Vigna radiata* (Linnaeus) R. Wilczek |  | Fabaceae | herb | cultivated | whole plant | decoction for medicinal bath | treat varicella | LC | 0.14 | 0.23 | YY210121005 |
| *Vitex negundo* Linnaeus |  | Lamiaceae | shrub | wild | branches | decoction for medicinal bath | treat rheumatism, promote blood circulation and remove blood stasis, relieve fatigue，runny nose, treat infantile common coldrunny nose, treat measles | LC | 0.58 | 0.91 | YY210601006 |
|  |  |  |  |  |  | decoction for oral administration | treat hypertension |  |  |  |  |
| *Vitex negundo* var. *cannabifolia* (Siebold & Zuccarini) Handel-Mazzetti |  | Lamiaceae | shrub | wild | branches | decoction for medicinal bath | relieve fatigue, relieve muscle pain, promote blood circulation and remove blood stasis, treat rheumatism, treat headaches | LC | 0.27 | 0.64 | HRC210907048 |
| Vitex quinata (Loureiro) Williams | wu zhi feng | Lamiaceae | tree | wild | branches | decoction for medicinal bath | help diaphoresis, treat rheumatism | LC | 0.08 | 0.00 | YY210119053 |
| *Wendlandia uvariifolia* Hance |  | Rubiaceae | tree | wild | branches | decoction for medicinal bath | remove dampness, stop itching and prevent infantile colds | LC | 0.35 | 0.82 | HRC210907034 |
| *Wikstroemia indica* (Linnaeus) C. A. Meyer | guan yin ma | Thymelaeaceae | shrub | wild | branches | decoction for medicinal bath | treat rheumatism | LC | 0.23 | 0.23 | YY210122004 |
| *Youngia japonica* (Linnaeus) Candolle |  | Asteraceae | herb | wild | whole plant | decoction for oral administration | clear heat in lungs | LC | 0.17 | 0.00 | YY210121032 |
| *Zanthoxylum nitidum* (Roxburgh) Candolle |  | Rutaceae | shrub | wild | root | decoction for medicinal bath | swelling and aching of gum | LC | 0.29 | 0.59 | YY210910034 |
| *Zanthoxylum avicennae* (Lam.) DC. | le dang mu | Rutaceae | tree | wild | root | decoction for oral administration | treat rheumatism, HBV | LC | 0.19 | 0.18 | YY201208003 |
